# Injectable Biomimetic Gels for Biomedical Applications

**DOI:** 10.3390/biomimetics9070418

**Published:** 2024-07-08

**Authors:** Hossein Omidian, Renae L. Wilson, Sumana Dey Chowdhury

**Affiliations:** Barry and Judy Silverman College of Pharmacy, Nova Southeastern University, Fort Lauderdale, FL 33328, USA; rw1273@mynsu.nova.edu (R.L.W.); sd2236@mynsu.nova.edu (S.D.C.)

**Keywords:** injectable hydrogels, tissue regeneration, biomimetic materials, controlled drug delivery, biocompatibility

## Abstract

Biomimetic gels are synthetic materials designed to mimic the properties and functions of natural biological systems, such as tissues and cellular environments. This manuscript explores the advancements and future directions of injectable biomimetic gels in biomedical applications and highlights the significant potential of hydrogels in wound healing, tissue regeneration, and controlled drug delivery due to their enhanced biocompatibility, multifunctionality, and mechanical properties. Despite these advancements, challenges such as mechanical resilience, controlled degradation rates, and scalable manufacturing remain. This manuscript discusses ongoing research to optimize these properties, develop cost-effective production techniques, and integrate emerging technologies like 3D bioprinting and nanotechnology. Addressing these challenges through collaborative efforts is essential for unlocking the full potential of injectable biomimetic gels in tissue engineering and regenerative medicine.

## 1. Introduction

Injectable biomimetic gels are a rapidly emerging class of materials designed to mimic the natural extracellular matrix (ECM) and facilitate tissue regeneration and repair. These hydrogels are engineered for minimally invasive delivery and in situ gelation, forming supportive scaffolds that promote cell growth, differentiation, and tissue integration. Their ability to conform to complex tissue geometries and provide localized, sustained release of therapeutic agents makes them highly versatile for various medical applications, including wound healing, drug delivery, and tissue engineering.

Despite their potential, injectable biomimetic gels face several challenges and limitations in biomedical research, necessitating ongoing research and development. A significant challenge is to enhance their adhesive and mechanical properties while ensuring biocompatibility and non-toxicity. Multifunctional gels need to exhibit sufficient bonding strength, self-healing capacity, and responsive adhesive abilities for effective wound closure and tissue repair [1,2,3,4,5]. These properties are crucial for the practical application of injectable gels in medical treatments.

One promising approach to address these challenges is the incorporation of protein-polysaccharide blends, inspired by the ECM, which benefit from the adhesive properties of proteins and the enhanced hydration and stiffness provided by polysaccharides. For instance, gelatin injectable hydrogels have shown improved mechanical properties when mixed with hyaluronic acid, enhancing the Young’s modulus and allowing tunable gelation times to meet specific surgical requirements [6]. Additionally, polysaccharide-based hydrogels, such as those combining calcium alginate with dextran methacrylate derivatives, have demonstrated synergistic mechanical behavior, which can be tailored for specific biomedical applications [7].

Achieving proper injectability and rapid gelation in situ is another essential requirement. Injectable hydrogels must maintain sufficient intrinsic adhesion and mechanical properties to support tissue regeneration. The ability to inject these materials easily and have them form gels quickly is vital for their use in minimally invasive procedures. Stimuli-sensitive injectable polymeric hydrogels, which can switch from sol-to-gel in response to various stimuli, offer promising solutions for controlled release and ease of handling, enhancing their applicability in drug delivery and tissue engineering [8,9,10,11].

Ensuring bioactivity and biocompatibility while maintaining mechanical integrity is fundamental for the success of injectable gels. Natural biopolymer-based hydrogels must support cell delivery, in situ biomineralization, and balance biological compatibility with mechanical performance [12,13,14,15]. These characteristics are necessary to mimic the natural environment of tissues and promote effective healing and regeneration. For example, 3D bioprinted scaffolds of natural-based hydrogels, incorporating proteins and polysaccharides, provide an excellent framework for cartilage tissue engineering by mimicking ECM structures and guiding cell growth [16].

Developing hydrogels that mimic natural cell environments and support vascular growth, bone regeneration, and tissue repair is a primary focus in this field. Hydrogels need to enhance structural and functional outcomes in regenerated tissues [17,18,19,20]. This includes creating materials that can closely replicate the properties of the tissues they are intended to replace or support. The creation of innovative composite materials that provide mechanical support and promote osteogenesis is another critical area of development. These materials need to be tough, injectable, and suitable for applications such as bone tissue engineering and osteoporotic fracture repair [21,22,23]. The ability to create materials that can support and encourage new bone growth while being easily applied via injection is crucial for their effectiveness.

Addressing advanced functionalities such as wound healing, antibacterial properties, and controlled drug delivery is also necessary. Hydrogels must be multifunctional, offering properties like self-healing, antimicrobial activity, and responsive delivery of therapeutic agents [24,25,26,27,28]. Polysaccharide-based bio-adhesives, particularly those using oxidized dextran and chitosan with dopamine for enhanced adhesion, exemplify advancements in creating injectable and sticky hydrogels suitable for tissue repair [29]. These functionalities are essential for the broad applicability of injectable gels in various medical treatments.

The field faces significant challenges related to enhancing adhesive and mechanical properties, achieving proper injectability and gelation, ensuring bioactivity and biocompatibility, supporting tissue regeneration and repair, developing innovative composite materials, and addressing advanced functionalities for specific medical applications. These collective concerns drive ongoing research and development to improve the efficacy and safety of injectable biomimetic gels.

## 2. Injectable Biomimetic Hydrogels

This section details different crosslinking methods used to enhance the properties and functionalities of these hydrogels. It also highlights specific types of hydrogels, including those based on gelatin, chitosan, alginate, hyaluronic acid, silk, collagen, and poly(ethylene glycol), describing their unique characteristics and applications in tissue engineering, regenerative medicine, and drug delivery.

Natural and synthetic-based hydrogels are increasingly being used in biomedical engineering, designed to mimic the natural healing processes of human tissues by being able to repair themselves after damage. Various crosslinking methods are employed to create these hydrogels, each contributing to their unique properties and functionalities. Key mechanisms include dynamic Schiff base reactions, which allow for reversible bonding and self-repair, and the use of aldehyde-terminated polymers that form stable networks. Additionally, borate and boronic ester bonds, as well as metal coordination and ionic crosslinking, provide adaptability and responsiveness to environmental changes. Physical crosslinking methods, such as hydrogen bonding and ion complexation, and chimerical crosslinking techniques also play a crucial role in enhancing the hydrogels’ structural integrity and responsiveness. Hydrophobic associations, used in thermoresponsive gels, and chain aggregation of polysaccharides and gums further contribute to the mechanical strength and flexibility of these materials. These methods collectively enhance the hydrogels’ integrity and mechanical properties and ability to release therapeutic agents in a controlled manner, making them highly effective for pharmaceutical and biomedical applications [30,31,32,33,34].

**Gelatin-Based Hydrogels:** Gelatin-based hydrogels are widely appreciated for their natural origin, biocompatibility, and ability to form gels at physiological temperatures, making them inherently biomimetic. These hydrogels provide a supportive environment similar to natural tissues, essential for cell growth and differentiation.

One advancement in this area has been the synthesis of a multifunctional injectable temperature-sensitive gelatin-based adhesive double-network hydrogel. This hydrogel combines strong adhesive properties with temperature sensitivity, making it suitable for applications requiring robust mechanical support and easy application through injection [1]. Similarly, dual crosslinking hydrogels engineered to mimic the ECM enhance cellular interactions and tissue regeneration, providing an ideal scaffold for regenerative therapies [12].

Innovative fabrication techniques have led to the development of EDC-crosslinked gelatin/nanohydroxyapatite injectable microspheres, which improve osteoconductivity, essential for bone tissue engineering [35]. Additionally, reinforcing gelatin-methacryloyl hydrogels with nanohydroxyapatite and nanosilicates enhances mechanical properties and bioactivity, making them versatile for various biomedical applications [9]. Bioinspired self-healing injectable nanocomposite hydrogels based on oxidized dextran and gelatin offer self-repair capabilities, crucial for maintaining long-term functionality in tissue engineering [14].

**Chitosan-Based Hydrogels:** Chitosan-based hydrogels, derived from chitin, are notable for their biocompatibility, biodegradability, and ability to form hydrogels with desirable properties for biomedical applications. These hydrogels can be easily injected, ensuring minimal invasiveness while providing a biomimetic environment conducive to tissue regeneration.

Engineered injectable cell-laden chitin/chitosan hydrogels support cell growth and tissue repair, making them valuable in regenerative medicine [36]. Thermosensitive chitosan-polygalacturonic acid polyelectrolyte complex hydrogels exhibit unique thermal responsiveness, adapting to body temperatures for effective in vivo applications [21]. The programmed release of VEGF and exosomes from chitosan nanofibrous microsphere-based PLGA-PEG-PLGA hydrogels showcases their potential in targeted and sustained therapeutic delivery [20].

Chitosan-based hydrogels also demonstrate enhanced mechanical strength and osteogenesis when incorporating nanoyarns for bone regeneration [37]. Genipin crosslinked bioactive collagen/chitosan/hyaluronic acid injectable hydrogels with silica particles combine multiple bioactive components, promoting tissue healing and regeneration [38]. Furthermore, self-healing properties in hydrogels based on oxidized alginate-hybrid-hydroxyapatite nanoparticles and carboxymethyl chitosan provide durability and efficient tissue repair [39].

**Alginate-Based Hydrogels:** Alginate-based hydrogels are derived from natural polysaccharides and are known for their biocompatibility and ability to form hydrogels under mild conditions, making them excellent biomimetic materials. These hydrogels can be injected into the body, providing a minimally invasive solution for tissue engineering and therapeutic applications.

The synthesis of bioconjugates by post-modification of alginate has led to advancements in bone tissue engineering, offering structural and functional support [13]. Bioinspired injectable self-healing hydrogel sealants based on alginate provide effective sealing and self-repair capabilities, ideal for wound management and surgical applications [2]. Additionally, mussel-inspired dual-functionalized alginate hydrogels exhibit strong adhesive properties, enhancing their versatility in medical applications [4].

Alginate-based hydrogels have also been developed for cartilage tissue engineering, where self-crosslinking and injectable properties ensure optimal performance and tissue integration [40]. The inclusion of functional components like nanocomposite materials, poly(L-glutamic acid), and alginate further enhances the mechanical and biological properties of these hydrogels, making them suitable for a wide range of therapeutic applications [41].

**Hyaluronic Acid-Based Hydrogels:** Hyaluronic acid-based hydrogels are particularly effective in cartilage and bone tissue engineering due to their natural biocompatibility and ability to mimic the ECM. These hydrogels are injectable, providing an easy and minimally invasive method for delivering therapeutic materials to target sites.

The fabrication of porous hyaluronic acid hydrogels allows for cell migration and nutrient exchange, which are essential for effective tissue regeneration [42]. Incorporating biphasic calcium phosphate microparticles into these hydrogels enhances their mechanical stability and osteoconductivity, making them ideal for bone repair [43]. Additionally, the development of self-crosslinking and injectable hyaluronic acid/RGD-functionalized pectin hydrogels provides an optimal environment for cartilage tissue engineering [44].

**Silk-Based Hydrogels:** Silk-based hydrogels, derived from silk fibroin, are known for their mechanical strength and biodegradability. These hydrogels are injectable and can be engineered to provide various biophysical and biochemical cues, making them highly versatile for biomedical applications.

Injectable hydrogels from silk fibroin and angiogenic peptides promote vascularization and tissue regeneration, making them suitable for wound healing and tissue engineering [17]. Autonomous self-healing silk fibroin hydrogels offer durability and long-term functionality, crucial for sustained therapeutic effects [45]. Additionally, the integration of silk fibroin with hydroxyapatite enhances their applicability in bone tissue engineering, providing necessary mechanical support and bioactivity [46].

**Collagen-Based Hydrogels:** Collagen-based hydrogels leverage the natural properties of collagen to support cell attachment and proliferation, making them ideal for soft tissue and bone regeneration. These hydrogels are injectable, allowing for minimally invasive delivery and easy application.

Injectable hydrogels incorporating nanoyarn structures demonstrate enhanced mechanical properties and support for bone regeneration [37]. Bifunctional hydrogels with photothermal effects offer innovative solutions for combined tumor therapy and bone regeneration, showcasing their versatility [47]. Furthermore, collagen-based hydrogels have been developed for specific applications like alveolar ridge preservation, providing tailored solutions for dental and orthopedic needs [48].

**Poly(ethylene glycol)-Based Hydrogels:** Poly(ethylene glycol) (PEG)-based hydrogels are known for their tunable properties and excellent biocompatibility. These hydrogels are injectable, making them suitable for various biomedical applications where minimally invasive methods are preferred.

Injectable biodegradable PEG/RGD peptide hybrid hydrogels facilitate in vitro chondrogenesis of human mesenchymal stem cells, highlighting their potential in cartilage repair [49]. The incorporation of lysozyme amyloid fibrils into PEG hydrogels improves antiswelling and antibacterial capabilities, expanding their utility in medical applications [28]. Additionally, PEG-based hydrogels have been developed for specific therapeutic applications, such as cardiomyocyte survival and maturation, showcasing their adaptability and functional benefits [50].

**Scheme 1:**
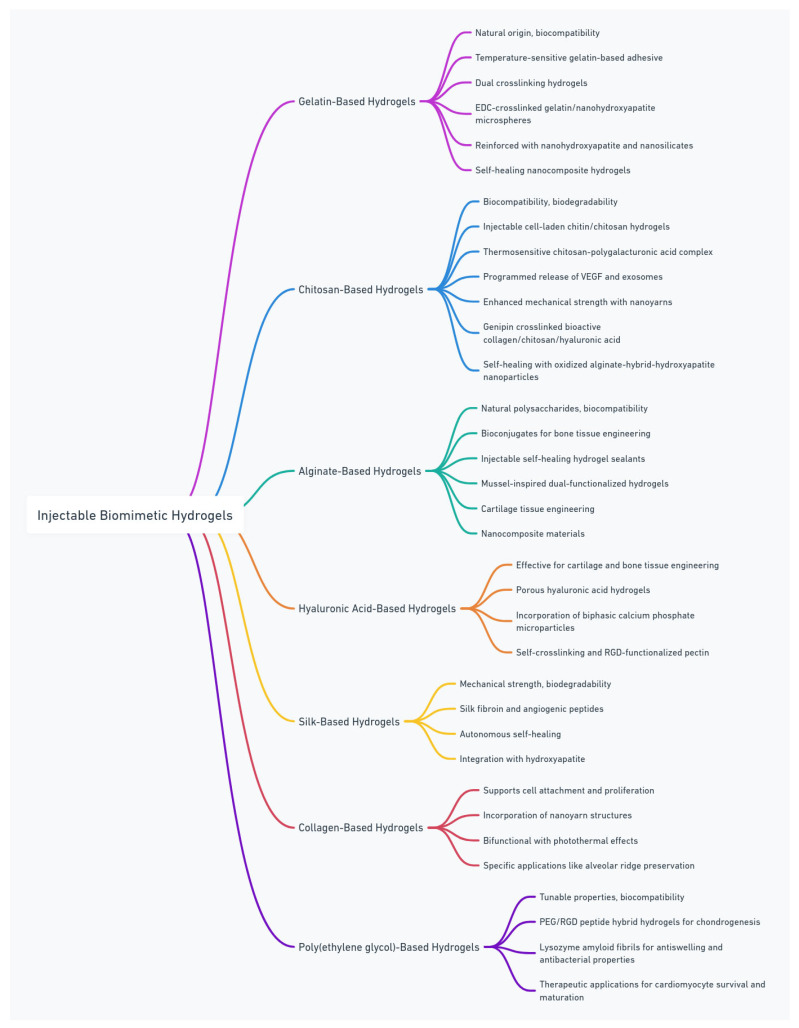
Structure–property relationships of injectable biomimetic gels.

The diverse array of polymers used in the development of injectable biomimetic hydrogels underscores their versatility and potential in biomedical applications. Each type of hydrogel offers unique properties and advantages, making them suitable for specific applications ranging from tissue engineering and regenerative medicine to drug delivery and wound healing. Scheme 1 visualizes the structure–property relationship for the most common injectable biomimetic gels.

## 3. General Regenerative Medicine

This section discusses various advancements in regenerative medicine, focusing on the development and application of innovative hydrogels. These hydrogels exhibit properties such as temperature sensitivity, mechanical flexibility, self-healing, antibacterial effects, and biocompatibility. Specific examples include gelatin-based adhesives, ECM-mimicking hydrogels, alginate bioconjugates, porous hyaluronic acid gels, and silk fibroin-peptide composites. Each type of hydrogel demonstrates unique advantages, such as promoting vascularization, supporting bone tissue engineering, and enhancing wound healing, making them valuable for diverse medical applications.

A gelatin-based adhesive double-network hydrogel has been developed utilizing catechol-Fe^3+^ and NIPAAm-methacryloyl, applied through a dual-syringe method. This DNGel exhibits significant temperature sensitivity, mechanical flexibility, strong adhesive strength, self-healing capabilities, antibacterial properties, and hemostasis promotion. These features are stabilized by molecular interactions between components in the DNGel [1].

Hydrogels mimicking the extracellular matrix (ECM) have been formulated using chondroitin sulfate and gelatin, with dual crosslinking via borate ester bonds and either Michael-addition or photopolymerization with thiol-containing PEG. This dual crosslinking strategy yields hydrogels with excellent injectability, mechanical resilience, and biocompatibility. The hydrogels feature tunable stiffness, resilience to compression, and effective energy dissipation, supporting cell encapsulation and 3D cell culture [12].

An alginate bioconjugate hydrogel incorporating poly(epsilon-caprolactone-co-lactide)-b-poly(ethylene glycol)-b-poly(epsilon-caprolactone-co-lactide) and O-phosphorylethanolamine has been studied for in situ gel formation, biomineralization, and sustained release of BMP-2 in vivo for bone tissue engineering. These alginate bioconjugate sols form stable gels at physiological temperatures, promote hydroxyapatite growth, and support BMP-2-loaded in situ biomineralization [13].

Porous hyaluronic acid (HA) hydrogels have been developed through an in situ bubble self-generation and entrapment process using cystamine dihydrochloride and EDC/NHS. The effects of concentration and viscosity on the hydrogel properties were investigated, revealing favorable biocompatibility. These HA hydrogels, formed via an amide reaction generating CO_2_ bubbles entrapped during gelation, showed a high elastic modulus and a porous structure. In vitro and in vivo studies demonstrated their biocompatibility and favorable cell behaviors [42].

**Figure 1:**
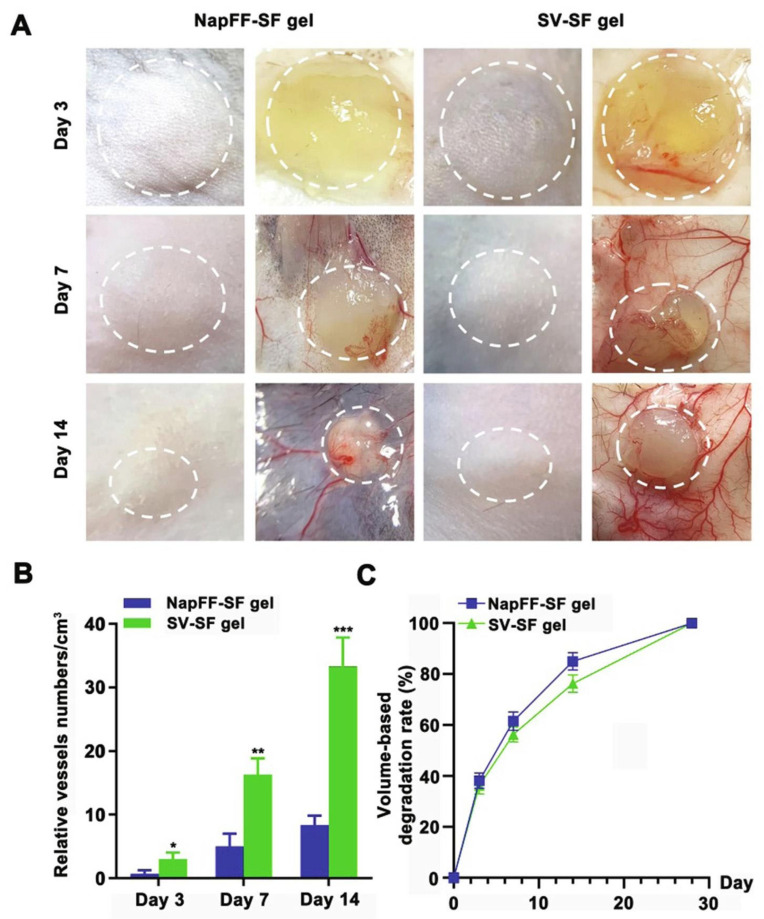
In vivo vascularization and degradation properties of the SV-SF hydrogel after subcutaneous injection into the dorsal side of mice. (**A**) External images and inside view of volume changes of the SV-SF and NapFF-SF hydrogels, and regenerated blood vessels within implanted gels and surrounding tissues on days 3, 7, and 14 after injection. (**B**) Summarized data about the relative number of new blood vessels regenerated within implanted gels and surrounding tissues on the dorsal side of mice within 14 days. (**C**) Summarized data about volume changes of the implanted SV-SF and NapFF-SF hydrogels on the dorsal side of mice within 28 days (*, *p* < 0.05, **, *p* < 0.01, ***, *p* < 0.001); adopted with permission [17].

A hydrogel composed of silk fibroin and the self-assembling peptide NapFFSVVYGLR has been described, which promotes endothelial cell adhesion, growth, and migration. The cooperative assembly of silk fibroin and NapFFSVVYGLR enhances vascularization and epidermal repair in mouse skin models. This SV-SF hydrogel exhibits good stability and induces endothelial cell adhesion, growth, and migration, thereby promoting vascularization and epidermal repair when implanted in mouse skin defects [17]. Figure 1 depicts the vascular regeneration and wound healing in vivo.

In another study, injectable and photo-curable hydrogels based on polymeric backbones modified for intrinsic adhesion and hybrid cross-linking were introduced. This bioinspired design strategy resulted in strong adhesive contact and a wide range of physicochemical properties. The adhesive networks, created via hybrid cross-linking, achieve a controlled synergy between interfacial chemistry and mechanical properties, making them suitable for applications such as tissue adhesives, surgical sealants, and injectable scaffolds [8].

Hypoxia preconditioned serum-fibrin (HPS-fibrin) hydrogels, incubated under hypoxic conditions to enhance their angiogenic potential, have been characterized for controlled growth factor delivery and the modulation of angiogenic responses. Hypoxic incubation increases the angiogenic potential of HPS; the fibrin hydrogels effectively retain and release HPS factors in a dose-dependent manner, thereby mimicking physiological wound healing processes [51].

## 4. Self-Healing and Responsive Hydrogels

This section highlights the development of self-healing and responsive hydrogels designed for advanced medical applications. These hydrogels exhibit properties such as injectability, self-healing, strong adhesion, antibacterial activity, and biocompatibility. Examples include hydrogels cross-linked through Schiff base bonds, dopamine-modified nanocomposites, catechol- and aldehyde-functionalized materials, and phenolic-chitosan composites. Each hydrogel type is evaluated for its mechanical properties, biodegradability, cytocompatibility, and effectiveness in wound healing, tissue adhesion, and cell development, demonstrating significant potential in medical treatments and tissue engineering.

**Figure 2:**
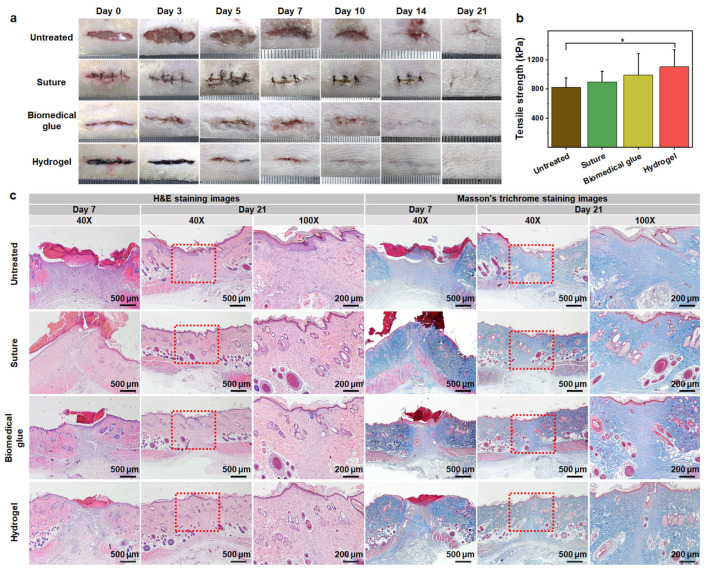
In vivo wound closure and healing evaluation. (**a**) Images of the incisions closed by suture, biomedical glue, adhesive hydrogel, and the wound without treatment (set as control). (**b**) The tensile strength of the healed skin tissues on day 21. (**c**) Images of H&E staining and Masson’s trichrome staining of the skin tissues after healing for 7 and 21 days. * *p*  <  0.05. Adopted with permission [2].

A hydrogel composed of sodium alginate, gelatin, protocatechualdehyde, and ferric ions was cross-linked via Schiff base bonds, catechol-Fe coordination bonds, and strong interactions between gelatin and sodium alginate. This hydrogel was evaluated for its potential in sutureless post-wound closure, injectability, self-healing capacity, repeated tissue adhesion, and its effectiveness in closing incisions in vivo. The hydrogel, stabilized through dynamic bonds, demonstrated excellent injectability, self-healing properties, repeated adhesion, photothermal antibacterial activity, and biocompatibility. It significantly promoted incision closure and wound healing in vivo (Figure 2) [2].

An injectable nanocomposite hydrogel was formed by combining dopamine-modified four-armed poly(ethylene glycol) with laponite. This hydrogel was assessed for its enhanced mechanical and adhesive properties. When implanted subcutaneously in rats, it exhibited minimal inflammatory response and improved cellular infiltration. The inclusion of up to 2 wt. % nanosilicate Laponite reduced the cure time and enhanced the mechanical and adhesive properties without altering the degradation rate or cytocompatibility, and resulted in improved bioactivity and mechanical properties [3].

A hydrogel prepared by grafting dopamine to aldehyde-modified alginate and crosslinking it with hydrazide-modified poly(l-glutamic acid) (PLGA-ADH) and dual-functionalized alginate (catechol- and aldehyde-modified alginate, ALG-CHO-catechol), was investigated for its mechanical properties, self-healing ability, adhesion, and hemostatic capability. This hydrogel was produced via a Schiff base reaction. The PLGA/ALG-CHO-catechol hydrogel exhibited enhanced mechanical properties, a reasonable gelation time, self-healing behavior, and superior hemostatic performance compared to its oxidized counterparts. It also demonstrated cytocompatibility [4].

A dynamic crosslinked hydrogel (DACS hydrogel) was created by mixing dopamine-grafted hyaluronic acid with carboxymethyl chitosan. This hydrogel was evaluated for its self-healing ability, biodegradation, biocompatibility, and wet tissue adhesion strength, making it a potential multifunctional tissue adhesive material. The gelation process was controlled by the degree of catechol substitution and the concentration of raw materials. The hydrogel exhibited rapid self-healing ability and a four-fold enhancement in wet tissue adhesion strength compared to commercial fibrin glue [5].

A phenolic-chitosan self-healing hydrogel was formed using N-[3-(4-hydroxyphenyl)propanamido] chitosan and a Pluronic-F127. This hydrogel, which developed hierarchical micelle architectures, was tested for thermoresponsiveness, adhesion to artificial skin, and the ability to embed mesenchymal stem cells for cell development. It demonstrated fast gelation within 30 s, thermoresponsiveness, strong adhesion to artificial skin, and supported the embedding and spheroid development of mesenchymal stem cells. The hierarchical micellar structures enhanced its overall functionality [52].

Chitosan grafted and crosslinked using l-glutamic acid, 1-ethyl-3-[3-dimethylaminopropyl]carbodiimide, and benzaldehyde-terminated four-arm poly(ethylene glycol) formed a hydrogel. This hydrogel was tested for biodegradability and cytocompatibility with the fibroblast cell line WI-38. It formed gel within 60 s, had a tunable compressive modulus, was degradable in PBS, and demonstrated cytocompatibility with fibroblast cells [53].

A hydrophobic-association hydrogel was formed by combining silk fibroin and stearyl methacrylate via reversible hydrophobic interactions. This hydrogel was tested for its self-healing properties, mechanical strength, degradability, biomimetic mineralization, and biocompatibility with mouse osteoblasts in vivo. The hydrophobic interactions served as sacrificial bonds that enhanced the mechanical properties and allowed self-recovery after injection. The hydrogel promoted a controlled degradation rate in a protease solution, supported biomimetic mineralization, and exhibited biocompatibility in vivo [45].

A gelatin hydrogel incorporating rod-shaped cellulose nanocrystals coated with magnetic nanoparticles, polydopamine, and poly(ethylene glycol) was tested under uniform low magnetic fields. The study evaluated its directional microstructure, anisotropic mechanical properties, and cellular performance with adipose tissue-derived human stem cells. Under magnetic fields, the nanoparticles within the gelatin hydrogels aligned to create directional microstructures and anisotropic mechanical properties. This alignment resulted in high cell viability and directional growth of the encapsulated adipose-derived human stem cells [18].

Hydrogels formed from zwitterionic copolymers (PMB) with benzoxaborole and zwitterionic glycopolymers (PMG) with varied sugar groups were created via benzoxaborole–sugar interactions. These hydrogels were assessed for their mechanical properties, self-healing capability, injectability, pH and sugar responsiveness, and biocompatibility. Rheological measurements and cytotoxicity tests confirmed that PMBG hydrogels, formed through dynamic interactions, had mechanical properties that could be tuned by varying the sugar content and pH. They demonstrated self-healing, injectability, and biocompatibility, as confirmed through in vitro cytotoxicity tests on both normal and cancer cells [54].

## 5. Bone Tissue Engineering

This section focuses on various natural polymers utilized in bone tissue engineering, such as hyaluronic acid, chitosan, gelatin, silk, and collagen. It details the development and evaluation of hydrogels based on these polymers, highlighting their composite formulations, mechanical properties, biodegradability, and effects on cell viability and osteogenesis. The hydrogels are tested both in vitro and in vivo, demonstrating their potential for applications like bone defect repair, angiogenesis, and bone regeneration. Additionally, differences in crosslinking methods and bioactive component integration are discussed to showcase their diverse functionalities and therapeutic potentials in bone tissue engineering

### 5.1. Natural Polymers: Hyaluronic Acid, Chitosan, Gelatin, Silk, Collagen

#### 5.1.1. Hyaluronic Acid-Based Hydrogels

A hyaluronic acid-g-chitosan-g-poly(N-isopropylacrylamide) hydrogel, integrated with biphasic calcium phosphate microparticles, has been characterized using X-ray diffraction and thermogravimetric analysis among other methods. This hydrogel was evaluated for its effects on cell viability, proliferation, gene expression, and osteoblastic differentiation utilizing human fetal osteoblast cells both in vitro and in vivo in nude mice. The HA-CPN/BCP hydrogel composite demonstrated an enhanced proliferation rate, increased alkaline phosphatase activity, and elevated gene expression in osteoblasts. Additionally, it successfully facilitated ectopic bone tissue formation when implanted subcutaneously in nude mice [43].

Hyaluronic acid/hydroxyapatite composite hydrogels were prepared using a Schiff base reaction with carboxymethyl chitosan. These hydrogels were assessed for their injectability, self-healing properties, cytocompatibility, and their ability to support cell adhesion and proliferation, making them suitable for bone defect repair. The inclusion of oxidized Hya/hydroxyapatite hybrid particles (OHAHs) in these hydrogels enhanced their mechanical properties and cytocompatibility, provided excellent cell adhesion, and supported proliferation in a 3D culture environment, confirming their suitability for injectable bone repair applications [55].

A hyaluronan-based hydrogel loaded with nano-hydroxyapatite crystals and bone morphogenetic protein-2 (BMP-2) was evaluated for its osteoinductive effects, particularly in enhancing bone density and architecture in the distal femur of both normal and ovariectomized New Zealand white rabbits. The Hya/HA composite hydrogel significantly improved bone architecture in both normal and ovariectomized rabbits, demonstrating strong osteoinductive effects and potential for enhancing bone density in osteoporosis [19].

#### 5.1.2. Chitosan-Based Hydrogels

A dual-drug releasing hydrogel composed of chitosan nanofibrous microspheres and poly(D,L-lactide-co-glycolide)-b-poly(ethylene glycol)-b-poly(D,L-lactide-co-glycolide) (PLGA-PEG-PLGA), loaded with vascular endothelial growth factor (VEGF) and dental pulp stem cell-derived exosomes, was developed to promote angiogenesis and osteogenesis. This hydrogel was tested in vitro and in vivo for bone formation in calvarial bone defects. The dual-drug releasing hydrogel demonstrated rapid VEGF release, promoting angiogenesis, and sustained exosome release, supporting osteogenesis. This significantly enhanced bone regeneration in calvarial bone defects [20].

An injectable chitin/chitosan hydrogel, cross-linked with acylhydrazone bonds and imine bonds, was combined with rat bone marrow mesenchymal stem cells (rBMSCs) for evaluation. The hydrogel’s mechanical strength, injectability, biodegradability, and ability to induce osteogenesis and support bone reconstruction in calvarial defects were assessed. This hydrogel, featuring dynamic imine and acylhydrazone bonds, exhibited robust mechanical strength, biodegradability, and self-healing properties. It supported rBMSC osteogenesis and effectively repaired bone defects over an eight-week period [36].

Chitosan and polygalacturonic acid polyelectrolyte complexes, integrated with beta-glycerophosphate and hydroxyapatite to form a thermosensitive hydrogel, were investigated for their biocompatibility, mechanical stability, osteogenic differentiation, and cellular responses using MG63 cells. These hydrogels, enhanced with gelatin and hydrothermally treated PEC fibers, demonstrated improved biocompatibility, compressive stiffness, and osteogenic activity, thereby supporting applications in bone tissue engineering [21].

Oxidized alginate hybrid hydroxyapatite nanoparticles and carboxymethyl chitosan hydrogel, prepared via a Schiff base reaction, were tested for their self-healing properties, cytocompatibility, and tunable gelling properties. These injectable hydrogels showed promising characteristics for bone tissue engineering applications, exhibiting self-healing capabilities, tunable gelation, and cytocompatibility. The hydrogels had a porous structure and well-distributed hydroxyapatite nanoparticles, making them suitable for bone repair [39].

#### 5.1.3. Gelatin-Based Hydrogels

Gelatin/nanohydroxyapatite composite microspheres crosslinked with 1-ethyl-3-(3-dimethylaminopropyl) carbodiimide have been characterized for their morphology, physicochemical properties, and cellular behavior. The EDC-crosslinked Gel-HA microspheres (ECM) demonstrated enhanced adhesion and growth of MG63 cells and showed sustainable bone regenerative effects in vivo. The Gel/n-HA composite microspheres exhibited promising morphology and physicochemical properties, along with effective bone repair in vivo, suggesting their potential as injectable microscaffolds for bone regeneration [35].

A gelatin-methacryloyl (GelMA) hydrogel loaded with mesenchymal stem cells (MSCs) and incorporating nanohydroxyapatite and nanosilicate was investigated for its osteogenic capacity both in vitro and in vivo. This hydrogel demonstrated enhanced cellular viability, proliferation, and bone regeneration in rat calvaria defects. The GelMA-HAP-SN hydrogel promoted MSC viability, proliferation, and osteogenic differentiation, resulting in significant bone regeneration in vivo, indicating its potential as an alternative to autologous bone grafts [9].

A composite of calcium phosphate cement (CPC) and gelatin methacryloyl (GelMA), combined with N-hydroxyethyl acrylamide (PHEAA), was formed through UV photo-initiation to create a GelMA-PHEAA hydrogel. This hydrogel was evaluated for its osteogenic potential, mechanical performance, and bioactivity in osteoporotic fracture healing. The GelMA-PHEAA and CPC composite hydrogels showed fast polymerization, enhanced mechanical properties, and significant osteogenic potential, making them promising candidates for clinical applications in osteoporotic fracture management [23].

**Figure 3:**
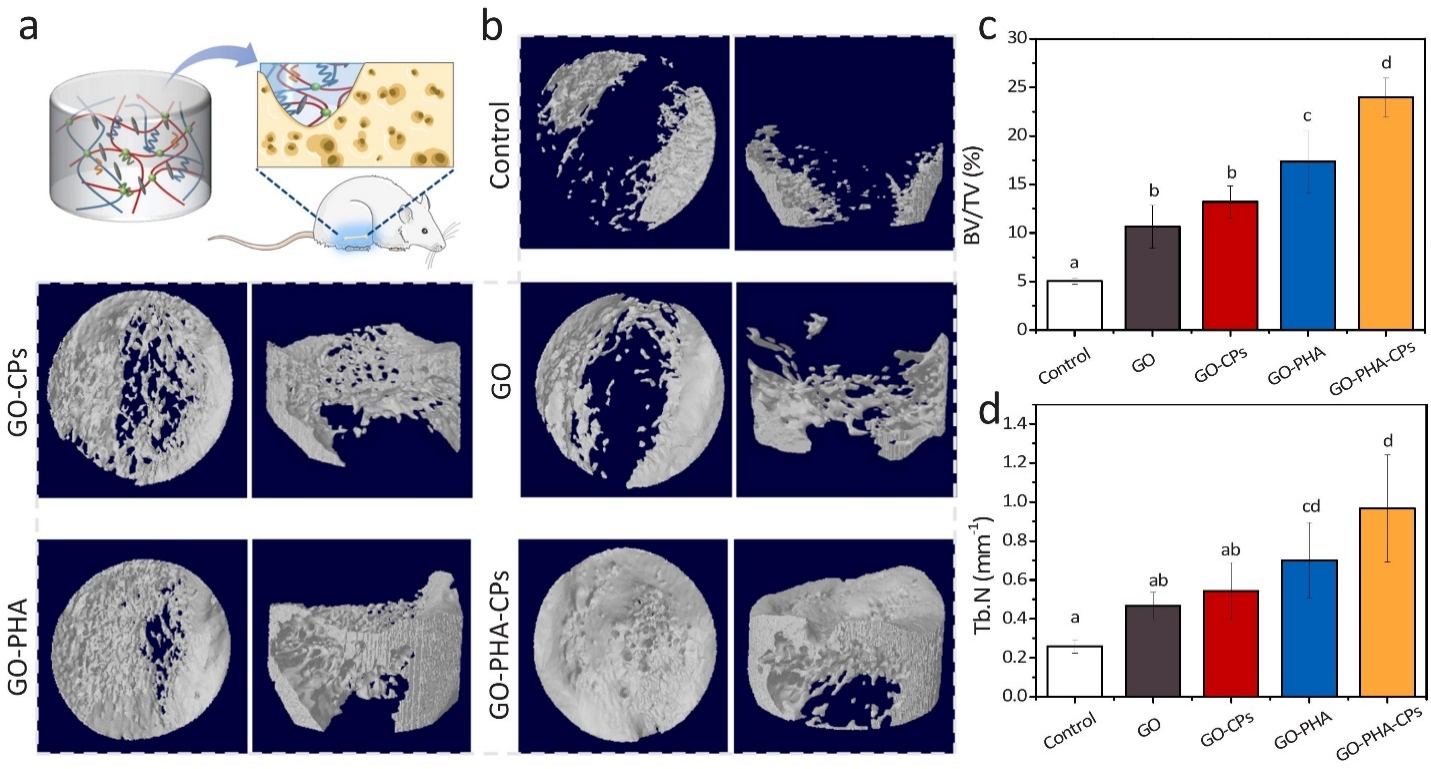
In vivo evaluation of bone regeneration. (**a**) Schematic representation of a rat femoral defect implanted with hydrogel. (**b**) Representative micro-CT images of the rat femoral bone defects at 5 weeks post-implantation. (**c**) Morphometric analysis of bone volume/tissue volume and (**d**) analysis of trabecular bone number. Adopted with permission [14].

A dopamine-modified gelatin (Gel-DA) and oxidized dextran (ODex) hydrogel containing polydopamine-functionalized nanohydroxyapatite (PHA) and cod peptides (CPs), all together termed as GO-PHA-CPs, was evaluated for its osteogenic activity, adhesion, and the spreading of MC3T3-E1 cells. This nanocomposite hydrogel supported the adhesion, spreading, and osteogenic differentiation of MC3T3-E1 cells, and it demonstrated enhanced bone regeneration in a rat femoral defect model [14]. Figure 3 demonstrates the in vivo evaluation of bone regeneration.

#### 5.1.4. Silk-Based Hydrogels

Injectable hydrogels made of silk nanofibers and hydroxyapatite nanoparticles, loaded with deferoxamine (DFO) and bone morphogenetic protein-2 (BMP-2), were assessed for their ability to promote angiogenesis and osteogenesis. These hydrogels regulated the delivery of DFO and BMP-2 and showed enhanced angiogenesis and osteogenesis, accelerating vascularized bone regeneration in cranial defects, achieving a composition and structure similar to natural bones [56].

Silk nanofiber hydrogels incorporating water-dispersible silk-hydroxyapatite nanoparticles were tested for their osteogenic properties, mechanical strength, and ability to heal bone defects in vivo. These composite hydrogels, with a high hydroxyapatite content (60% *w*/*w*) and a modulus of approximately 21 kPa, induced osteodifferentiation and demonstrated improved osteogenesis compared to silk nanofiber hydrogels, effectively healing bone defects in vivo [46].

Dense collagen hydrogels with silk sericin were prepared by aspirating and ejecting highly hydrated collagen gels and incorporating silk-extracted sericin, along with negatively charged protein rich in acidic amino acids (Asp and Glu). These hydrogels were investigated for hydroxyapatite deposition in simulated body fluid, and the proliferation and osteogenesis of mesenchymal stem cells (MSCs) in vitro. The incorporation of silk sericin in the dense collagen gels increased hydroxyapatite deposition and osteogenic markers in MSCs, accelerating cell-induced mineralization, suggesting their potential for bone tissue engineering applications [57].

#### 5.1.5. Collagen-Based Hydrogels

Hydrogels based on collagen, chitosan, and hyaluronic acid crosslinked with genipin were tested for their mechanical properties, degradation rate, wettability, and biocompatibility. These hydrogels supported the proliferation and adhesion of MG-63 cells, making them promising candidates for bone regeneration applications. They exhibited good mechanical properties, prolonged degradation, and biocompatibility, supporting cell proliferation and adhesion [58].

Collagen/chitosan/hyaluronic acid-based hydrogels incorporating amino-functionalized silica particles and crosslinked with genipin were evaluated under simulated body fluid conditions and in vitro cell culture. These hydrogels demonstrated bioactivity and biocompatibility, supporting the adhesion of MG-63 cells, proliferation, and alkaline phosphatase (ALP) expression. The covalently attached amino-functionalized silica particles enhanced the bioactivity and biocompatibility of the biopolymeric network [38].

Poly(L-lactide-co-epsilon-caprolactone) nanoyarns were suspended in a type I collagen hydrogel to enhance its mechanical properties. This composite supported the proliferation of human mesenchymal stem cells (hMSCs) and showed higher alkaline phosphatase activity and osteocalcin expression, indicating osteogenic differentiation. The incorporation of P(LLA-CL) nanoyarns improved the mechanical properties and injectability of the collagen hydrogel, effectively supporting hMSC proliferation and osteogenic differentiation [37].

A collagen-hyaluronic acid hydrogel embedded with calcium sulfate nanorods was evaluated for its self-biomineralization capabilities, cell adhesion, and proliferation. This composite hydrohel CSN@Col-HA facilitated self-biomineralization, quickly forming bone-like hydroxyapatite and stimulating preosteoblast differentiation. The hydrogel promoted in situ bone growth and hydroxyapatite formation in bone defects, suggesting its potential for minimally invasive bone regeneration [59].

### 5.2. Synthetic Polymers and Composites

A triblock copolymer hydrogel composed of PEG-PCL-PEG, collagen, and nano-hydroxyapatite was characterized for its microstructure, thermo-responsiveness, biocompatibility, and biodegradability. This composite hydrogel (PECE/Collagen/n-HA) was evaluated for guided bone regeneration in cranial defects of rabbits in vivo. It exhibited an interconnected porous structure, good thermo-sensitivity, and biodegradability. In vivo studies confirmed its biocompatibility and enhanced osteogenic capacity in cranial defects of rabbits over periods of 4, 12, and 20 weeks [60].

Injectable hydrogels composed of MgO/MgCO_3_@PLGA formed porous scaffolds with controllable magnesium ion release. These hydrogels were tested for their injectability, osteogenic differentiation, biomineral deposition, and bone regeneration in rat calvarial defects. The PMM hydrogels transformed into porous scaffolds in situ, filled irregular defects, and sustainably released Mg^2+^, enhancing cell proliferation, osteogenic differentiation, and biomineral deposition. This effectively stimulated in situ bone regeneration, increasing bone volume fraction and shape adaptability [61].

A composite hydrogel (EPH) consisting of enzyme-catalyzed amorphous biomineral nanoparticles, fibrins, and platelets was evaluated for its biocompatibility, osteogenic differentiation capabilities in rat bone marrow stem cells in vitro, and its effectiveness in promoting bone regeneration in vivo. This hydrogel demonstrated high biocompatibility and superior bioactivity in promoting osteogenic differentiation of rBMSCs. In vivo studies indicated that the EPH hydrogel significantly enhanced new collagen and vessel formation, accelerating bone defect regeneration within two weeks [62].

### 5.3. PMMA and Functionalized Hydrogels

A poly(methyl methacrylate) (PMMA) composite integrated with chitosan-glycerophosphate thermosensitive hydrogel, nano-hydroxyapatite, and gentamicin was developed to improve PMMA performance for bone tissue applications. This composite exhibited open pores, reduced polymerization temperature, enhanced antibacterial activity, and improved mineralization capacity, facilitating better bone tissue ingrowth. The CS-GP hydrogel augmented PMMA cement by providing superior bone tissue integration, lowering polymerization temperatures, and offering appropriate mechanical properties. Additionally, it increased mineralization capacity and demonstrated enhanced antibacterial properties [63].

A functionalized PMMA cement combined either with nano-hydroxyapatite, silver ions, or a combination of both, also incorporating chitosan-poly(vinyl alcohol) (CS-PVA) hydrogel, was tested for its physicochemical properties, antibacterial activity, biomineralization ability, and mechanical strength. This composite p-PMMA/CS-PVA/Nano-HA/Ag^+^ showed promising results for bone reconstruction applications, with improved mechanical properties, enhanced mineralization, and increased antibacterial activity. The functionalized PMMA cement demonstrated potential as an injectable, multi-functional bone cement for clinical use [22].

### 5.4. Miscellaneous Hydrogels with Unique Compositions

Methylcellulose hydrogels incorporating calcium phosphate (CaP) or calcium phosphate modified with graphene oxide (CaPGO) formed thermo-responsive biocomposites. These hydrogels were evaluated for their injectability, mineralization in simulated body fluid, cell adhesion, proliferation, and osteogenic differentiation, with characterization by X-ray diffraction and nuclear magnetic resonance. The hydrogels with CaP and CaPGO retained their injectability, promoted in vitro mineralization, and enhanced cell growth and osteogenic differentiation. The biomimetic phase comprised both crystalline hydroxyapatite and amorphous calcium phosphate, indicating their potential for bone tissue engineering [10].

An injectable nanocomposite hydrogel composed of gelatin, alginate, and laponite, loaded with rat bone marrow mesenchymal stem cells (rBMSCs), was found to promote cell proliferation in vitro and significantly enhance bone healing in critical-size rat calvarial defects in vivo. This nanocomposite hydrogel mimicked the extracellular matrix architecture, supported rBMSC survival and proliferation, and significantly enhanced bone regeneration in vivo, making it a promising candidate for clinical orthopedic applications [64]. A hybrid hydrogel composed of laponite and calcium phosphate incorporated into gelatin (LC hydrogel) was designed with a controlled degradation rate. This hydrogel was assessed for its osteoinduction, osteoconduction, angiogenesis, and ability to transform into new bone, supporting ligament graft osseointegration in vivo. The LC hydrogel demonstrated a degradation rate compatible with bone regeneration, promoting osteoinduction, osteoconduction, and angiogenesis. This facilitated functional bone regeneration and skeletal muscle repair, highlighting its suitability for minimally invasive therapeutic applications [65].

An injectable hydrogel crosslinked via a Schiff base reaction between the aldehyde groups on oxidized sodium alginate and amino groups on chitosan, containing cisplatin and polydopamine-decorated nano-hydroxyapatite, was evaluated for tumor therapy and bone regeneration. This hydrogel demonstrated photothermal effects, sustained drug release, and promoted bone mesenchymal stem cell adhesion and proliferation. It exhibited effective tumor cell ablation through photothermal effects and enhanced bone regeneration by supporting BMSC proliferation and adhesion [47].

### 5.5. Similarities and Differences between Gels Used in Bone Tissue Engineering

The methodologies for developing injectable biomimetic gels in bone tissue engineering share several commonalities. Firstly, they often employ natural polymers like hyaluronic acid, chitosan, and gelatin due to their biocompatibility and ability to support cell proliferation and differentiation. For instance, hyaluronic acid-based hydrogels have been combined with biphasic calcium phosphate microparticles to enhance osteogenic differentiation and ectopic bone tissue formation [43]. Similarly, chitosan-based hydrogels are utilized for their biodegradability and osteogenic capabilities [20,36]. Most studies incorporate bioactive components such as hydroxyapatite, bone morphogenetic proteins (BMPs), and vascular endothelial growth factor (VEGF) to promote bone regeneration and angiogenesis [19,20,56]. These hydrogels are often evaluated for their mechanical properties, injectability, biodegradability, and cell-supportive characteristics in both in vitro and in vivo settings [14,55].

For each hydrogel composition, however similar, the methodologies, specific polymer combinations, and crosslinking strategies used can differ. For instance, while some hyaluronic acid-based hydrogels use a Schiff base reaction with carboxymethyl chitosan to enhance mechanical properties and cytocompatibility [55], others integrate nano-hydroxyapatite and BMP-2 for their osteoinductive effects [19]. Chitosan-based hydrogels also exhibit variations; some use acylhydrazone bonds and imine bonds for crosslinking to achieve self-healing properties [36], whereas others incorporate polygalacturonic acid and beta-glycerophosphate to form thermosensitive hydrogels [21]. Gelatin-based hydrogels differ in their crosslinking agents and additional components, such as methacryloyl modifications and incorporation of nanosilicate or calcium phosphate to enhance osteogenic capacity [9,23]. Synthetic polymers and composites, like the triblock copolymer PEG-PCL-PEG, introduce thermo-responsiveness and biodegradability, distinguishing them from natural polymer-based hydrogels [60]. Furthermore, some methodologies focus on multifunctional approaches, incorporating antibacterial and photothermal therapy capabilities in addition to bone regeneration [22,47].

## 6. Cartilage Tissue Engineering

Thise section discusses various hydrogels and their potential for cartilage regeneration. It highlights different hydrogel types, including alginate-based, methacrylate-based, hyaluronic acid-based, chondroitin sulfate-based, and silk-based hydrogels. These hydrogels are evaluated for their biocompatibility, mechanical properties, degradation rates, and ability to support chondrocyte attachment, proliferation, and chondrogenesis. The studies demonstrate the hydrogels’ potential for pharmaceutical delivery, 3D bioprinting, injectable cartilage repair, and cartilage tissue engineering applications, showcasing their adaptability and effectiveness in promoting cartilage formation and repair.

### 6.1. Alginate-Based Hydrogels

An oxidized alginate/gelatin hydrogel self-crosslinked with borax was evaluated for its potential in cartilage tissue engineering. This hydrogel, which formed within 20 s, demonstrated excellent adhesive properties and negligible inflammatory responses. It supported the attachment, proliferation, and maintenance of the phenotype of primary murine chondrocytes. The hydrogel also exhibited tunable glycosaminoglycan (GAG) deposition and DNA content, indicating its potential for neo-cartilage formation [40].

A hydrogel composed of poly(L-glutamic acid) (PLGA) and alginate, crosslinked via the self-crosslinking of hydrazide-modified PLGA and aldehyde-modified alginate, was investigated for its gelation time, swelling behavior, mechanical properties, degradation rate, cell viability, and in vivo cartilage formation capabilities. The PLGA/ALG hydrogels demonstrated good biocompatibility and mechanical stability, supporting the viability of encapsulated chondrocytes, rapid gel formation in vivo, and promoting cell ingrowth and cartilage formation. These properties suggest its suitability for pharmaceutical delivery and tissue engineering applications [41].

### 6.2. Methacrylate-Based Hydrogels

A methacrylated cartilage extracellular matrix-based hydrogel (cECM-MA) photocrosslinked with UV light was characterized for its rheological properties, suitability for bioprinting, and chondrogenesis. Bioinks loaded with bone marrow-derived mesenchymal stem cells (BM-MSCs) generated 3D constructs that supported cell viability and chondrogenesis. The cECM-MA bioinks exhibited shear-thinning properties, were photocrosslinkable, and maintained BM-MSC viability and chondrogenesis post-printing, indicating their potential for 3D bioprinting and injectable cartilage tissue engineering [66].

A hydrogel composed of gelatin methacrylate (GELMA) and poly(ethylene glycol) diacrylate (PEGDA), light-cured with UV light and modified with kartogenin (KGN), was tested for its mechanical properties, degradation rate, and ability to repair cartilage in full-thickness osteochondral defects. The GELMA/PEGDA + KGN scaffolds demonstrated increased mechanical properties, prolonged degradation, and induced differentiation of endogenous stem cells into chondrocytes, effectively repairing cartilage defects and restoring cartilage to hyaline cartilage [67].

A locust bean gum-methacrylate (LBG-MA) hydrogel, photocrosslinked via UV irradiation, was evaluated for its degradation rate, mechanical properties, biocompatibility, chondrogenic differentiation, and cartilage healing in vivo. The LBG-MA hydrogels exhibited controllable degradation, improved mechanical properties, and excellent biocompatibility. They significantly induced the chondrogenic differentiation of bone mesenchymal stem cells (BMSCs) and promoted cartilage regeneration after 8 weeks of therapy [68].

**Figure 4:**
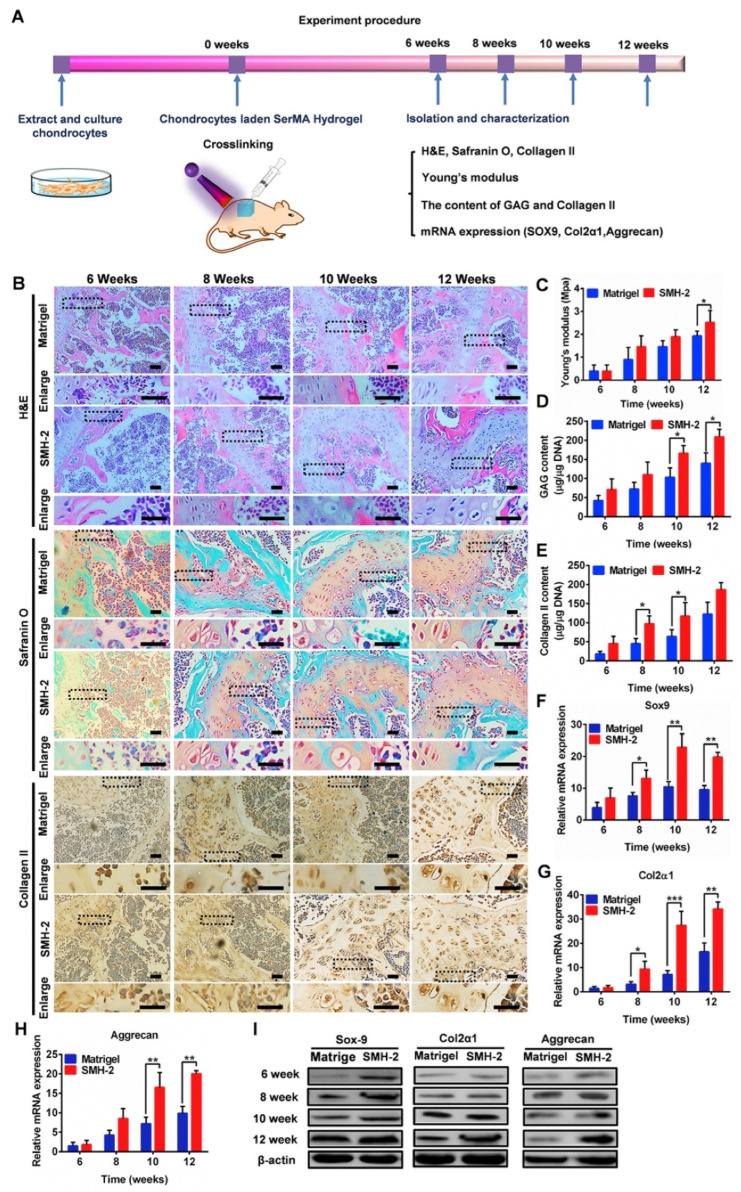
Artificial cartilage formation in vivo. (**A**) The experimental procedure for injectable SMHs for forming cartilage in vivo. (**B**) Histochemical staining of chondrocyte-embedded SMH-2 and Matrigel implanted subcutaneously in nude mice. Top, hematoxylin and eosin (H&E) staining; middle, safranin O staining; bottom, collagen II staining. The black line dotted boxes in the upper panel were enlarged in the lower panel. Scale bars, 50 μm. (**C**) The Young’s moduli of formed artificial cartilages. (**D**,**E**) The GAG (D) and collagen II (**E**) content in chondrocytes cultured within SMH-2 and Matrigel after 6, 8, 10, and 12 weeks. (**F**–**H**) The relative mRNA levels of Sox9 (**F**), Col2α1 (**G**), and Aggrecan (**H**) in chondrocytes from SMHs after a 12 week implantation in vivo. (**I**) The protein expression of chondrogenic genes (SOX-9, Col2α1, and Aggrecan) in chondrocytes from SMHs during a 12 week implantation in vivo. * *p* < 0.05, ** *p* < 0.01, *** *p* < 0.001; *n* = 3 per group per condition. Adopted with permission [69].

A photo-crosslinkable sericin methacryloyl (SerMA) hydrogel that formed pure sericin hydrogel (SMH) was evaluated for its biocompatibility, chondrocyte adhesion and proliferation, mechanical properties, degradation rate, and cartilage repair in vivo. The SMH hydrogels demonstrated excellent biocompatibility, supported chondrocyte adhesion and proliferation, and had tunable mechanical properties and degradation rates. They effectively formed artificial cartilages in vivo after 8 weeks, suggesting their potential for cartilage tissue engineering (Figure 4) [69].

### 6.3. Hyaluronic Acid-Based Hydrogels

Hyaluronic acid-adipic dihydrazide and oligopeptide G(4)RGDS-grafted oxidized pectin hydrogel, crosslinked via hydrazone bonds, was evaluated for its physicochemical properties, chondrocyte behavior, and tissue compatibility using a mouse subcutaneous implantation model. This hydrogel supported the phenotype of chondrocytes and facilitated chondrogenesis, demonstrating their potential as biomaterial scaffolds for cartilage regeneration [44].

A hydrogel created by grafting hyaluronic acid with a dextran-tyramine conjugate and enzymatically crosslinked with horseradish peroxidase and hydrogen peroxide was assessed for gelation, swelling, mechanical properties, chondrocyte viability, proliferation, and matrix production. The HA-g-Dex-TA hydrogels formed rapidly and supported chondrocyte viability, proliferation, and matrix production, indicating their suitability as injectable scaffolds for cartilage tissue engineering [70].

Hyaluronic acid-based hydrogels incorporating calcium or phosphate components and alginate, forming interpenetrating networks, were evaluated for their stress relaxation, mechanical properties, biphasic chondrogenesis, and cartilage regeneration in vivo. These HA-based hydrogels with calcium or phosphate components demonstrated stress relaxation, self-healing, and shear-thinning properties, promoting biphasic chondrogenesis and showing potential for mimicking natural cartilage [71].

A di-self-crosslinking hyaluronan-based hydrogel combined with type I collagen, crosslinked via thiol/maleimide click chemistry and thiol oxidation, was investigated for its injectability, mechanical properties, chondrocyte adhesion, proliferation, and cartilage formation in vivo. The HSMSSA/Col I hydrogels exhibited improved mechanical properties, enhanced chondrocyte adhesion and proliferation, and promoted cartilaginous tissue formation in vivo, suggesting their potential as injectable fillers for cartilage repair [72].

### 6.4. Chondroitin Sulfate-Based Hydrogels

Hydrogels composed of carboxymethylated pullulan-tyramine and chondroitin sulfate-tyramine, enzymatically crosslinked with horseradish peroxidase and hydrogen peroxide, were evaluated for their mechanical stability, cytocompatibility, chondrogenesis, and tissue compatibility using porcine auricular chondrocytes and a mouse subcutaneous implantation model. The CMP-TA/CS-TA composite hydrogels demonstrated good physicochemical properties, supported chondrocyte proliferation and extracellular matrix (ECM) deposition, and exhibited acceptable tissue compatibility in a mouse subcutaneous implantation model, indicating their potential for cartilage regeneration [73].

A hydrogel combining chondroitin sulfate with hyperbranched multifunctional poly(ethylene glycol) (HB-PEG), crosslinked via thiol-ene reaction, was evaluated for mechanical properties, degradation, and chondrogenesis using rat adipose-derived mesenchymal stem cells. The CS-SH/HB-PEG hydrogels demonstrated rapid gelation, excellent mechanical properties, prolonged degradation, and supported the viability and chondrogenesis of adipose-derived mesenchymal stem cells with a reduced inflammatory response, making them promising for cartilage tissue engineering [74].

A hydrogel functionalized with chondroitin sulfate, adipic dihydrazide, and oxidized pullulan, crosslinked via hydrazone bonds, was investigated for gelation time, swelling behavior, degradation, mechanical properties, and chondrogenesis using rabbit articular chondrocytes. The CS-ADH/oxPL hydrogels exhibited good gelation, swelling, degradation behavior, and mechanical properties, supporting chondrocyte encapsulation and cartilaginous ECM deposition, indicating their potential as injectable scaffolds for cartilage tissue engineering [75].

A hydrogel formed by combining collagen type II and activated chondroitin sulfate (sNHS) was investigated for its gelation time, water absorption, mechanical properties, chondrocyte survival, proliferation, and ECM remodeling. The collagen type II and CS-sNHS hydrogels formed fibrous structures that supported chondrocyte viability, proliferation, and ECM remodeling, demonstrating their potential as injectable carriers for chondrocyte delivery in cartilage tissue engineering [76].

A bilayer scaffold was developed using collagen-carbonyl hydrazide (COL-CDH) and oxidized chondroitin sulfate (OCS) with poly(ethylene glycol) diacrylate (PEGDA) functioning as a cartilage layer, and zinc-doped hydroxyapatite for the subchondral bone layer. This scaffold was investigated for its ability to support adipose mesenchymal stem cell differentiation, glycosaminoglycan secretion, calcium deposition, and regeneration of cartilage and subchondral bone in vivo. The bilayer hydrogel scaffold demonstrated strong interface binding, supported stem cell differentiation, and enhanced glycosaminoglycan and calcium deposition, thereby improving osteochondral repair [77].

### 6.5. Silk-Based Hydrogels

A silk fibroin hydrogel combined with sulfated-carboxymethyl cellulose (s-CMC) and tyraminated-carboxymethyl cellulose (t-CMC) was crosslinked using horseradish peroxidase and hydrogen peroxide. This hydrogel was designed to mimic the extracellular matrix (ECM) by providing gradual stiffening and presenting growth factors. The s-CMC/t-CMC/silk IPN hydrogels promoted chondrogenic differentiation of stem cells and supported cartilage ECM deposition, effectively mimicking the natural ECM [78].

Methacrylate-modified silk fibroin hydrogels embedded with kartogenin (SilMA/KGN) were evaluated for their biohistocompatibility, ability to promote cartilage differentiation, and potential for superficial cartilage repair in vivo. These hydrogels exhibited good biohistocompatibility, enhanced cartilage differentiation, and supported cell adhesion and proliferation. Significant superficial cartilage repair was observed in vivo, indicating their potential for cartilage tissue engineering [79].

### 6.6. Other Hydrogels

A hybrid hydrogel composed of poly(ethylene glycol) (PEG) and arginine-glycine-aspartic peptide (RGD) was crosslinked via Michael addition and assessed for cell viability, chondrogenesis, and type II collagen expression using human mesenchymal stem cells (hMSCs). The PEG-RGD hydrogels with a concentration of 1.0 mM RGD and TGF-beta3 showed significantly higher expression of type II collagen and positive staining for aggrecan and type II collagen, making them promising scaffolds for delivering hMSCs for cartilage repair [49].

A chitosan-based hydrogel incorporating articular cartilage stem cells (ACSCs) and mesoporous silica nanoparticles loaded with anhydroicaritin (AHI) was investigated for its sustained release properties, ACSC proliferation and differentiation, ECM production, and cartilage regeneration in vivo. This injectable nanocomposite hydrogel with sustained release of AHI improved ACSC chondrogenesis and cartilage regeneration, functioning effectively as a 3D biomimetic extracellular matrix [80].

A hydrogel composed of polyamidoamine dendrimer G3, mesoporous silica nanoparticles, and dendrimer-templated silver nanoparticles was crosslinked with poly(ethylene glycol) diglycidyl ether. This hydrogel was tested for its elasticity, mechanical properties, dual drug release (isoniazid and rifampicin), injectability, swelling capacity, structural stability, and cytocompatibility. This multifunctional hydrogel (MBGs with G3, MSN-NH2, and G3-Ag) demonstrated enhanced mechanical properties, dual drug release capabilities, and cytocompatibility, indicating its potential as an injectable gel graft for cartilage defect repair and antibiotic delivery [81].

Glycopolypeptide hydrogels were synthesized from poly(gamma-propargyl-L-glutamate) conjugated with azido-modified mannose and 3-(4-hydroxyphenyl) propanamide using click chemistry. Injectable hydrogels were developed using this glycopolypeptide through an enzymatic crosslinking reaction with horseradish peroxidase (HRP) and hydrogen peroxide (H_2_O_2_). These hydrogels were evaluated for their physicochemical properties, biocompatibility, chondrocyte viability, proliferation, and cartilage matrix production. The glycopolypeptide hydrogels exhibited controlled physicochemical properties, good cytocompatibility, and enhanced production of glycosaminoglycans (GAG) and type II collagen, maintaining the chondrocyte phenotype in subcutaneous models, suggesting their potential as scaffolds for cartilage tissue engineering [82].

### 6.7. Similarities and Differences between Gels Used in Cartilage Tissue Engineering

Various research studies on injectable biomimetic gels for cartilage tissue engineering have revealed several similarities in their methodologies. A common approach involves using naturally derived or synthetic polymers to form hydrogels, often modified to improve their biocompatibility, mechanical properties, and degradation rates. For instance, alginate-based hydrogels are crosslinked with various agents like borax [40] or hydrazide-modified PLGA [41], while methacrylate-based hydrogels utilize UV light for photocrosslinking [66,67,68,69]. Hyaluronic acid-based hydrogels also employ crosslinking strategies such as enzymatic crosslinking with horseradish peroxidase and hydrogen peroxide [70]. Across these studies, hydrogels are assessed for their chondrocyte viability, proliferation, and ability to support chondrogenesis, indicating a standardized approach towards evaluating the potential of these materials in cartilage regeneration.

There are similarities and notable differences that exist in the methodologies of these studies. The types of polymers and crosslinking methods vary significantly. For example, alginate-based hydrogels often use self-crosslinking techniques [40,41], whereas methacrylate-based hydrogels rely on photoinitiated crosslinking [66,67,68,69]. Hyaluronic acid-based hydrogels differ further by incorporating components like calcium or phosphate to form interpenetrating networks [71], and chondroitin sulfate-based hydrogels use enzymatic or chemical crosslinking [73,74,75,76,77]. Additionally, the evaluation metrics differ; some studies focus on mechanical properties and degradation rates [67,69,72], while others emphasize biological responses such as cell viability and chondrogenic differentiation [40,66,68,70].

## 7. Soft Tissue Engineering, Meniscus, and Myocardial Applications

The section delves into various hydrogel applications in soft tissue engineering, meniscus repair, and myocardial tissue regeneration. It details the composition and evaluation of different hydrogels, such as PIC hydrogels with growth factors for abdominal wound healing, SF/HA hydrogels for cartilage regeneration, and glycopeptide hydrogels for vascularization. Additionally, it discusses PVA and silk fibroin hydrogels for meniscus engineering and PHEMA-based hydrogels for myocardial applications. The overarching goal of these studies is to create biomimetic scaffolds that enhance tissue repair by improving mechanical properties, cell viability, and integration.

**Figure 5:**
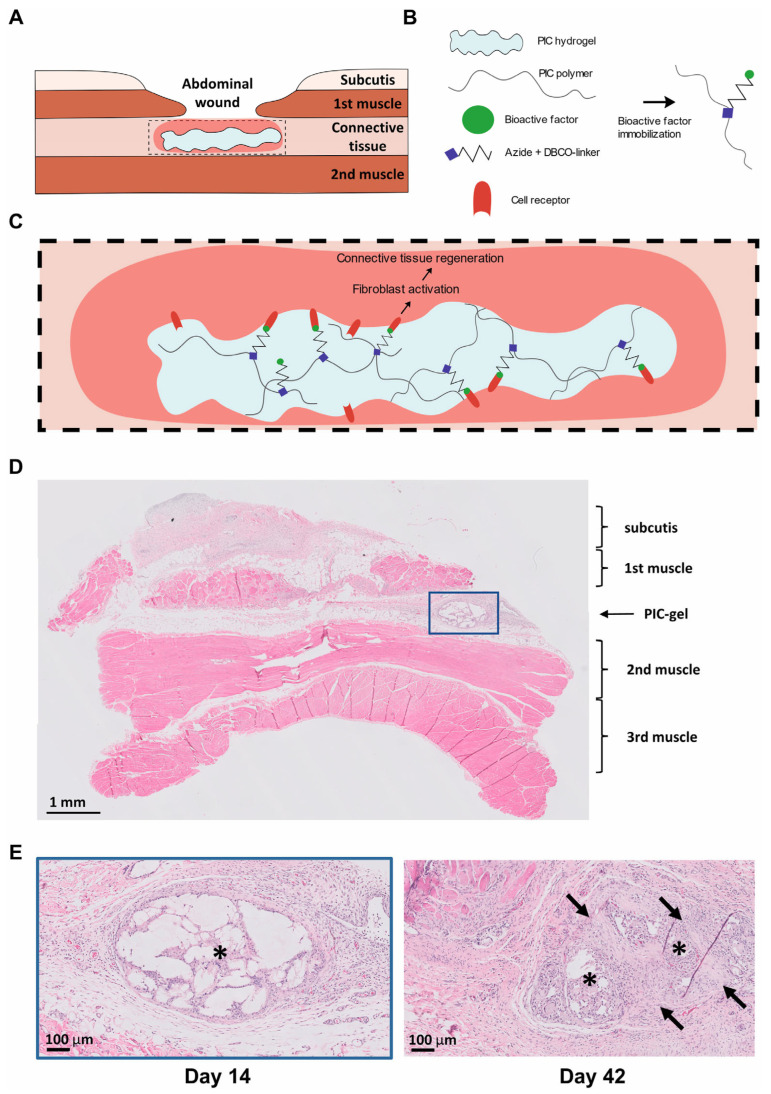
Schematic illustrations and histological images of a regenerative strategy with the use of polyisocyanide (PIC) hydrogel for application in abdominal connective tissue. (**A**) The application of the hydrogel to the abdominal wound site. (**B**) PIC-bFGF and PIC-E2, immobilization via azide and the DBCO-linker for the delivery of biomolecules to the tissues. (**C**) A schematic illustration showing the interaction between the factors and tissue that should trigger regeneration. (**D**) A histology image of H&E stains confirming the presence of PIC gel between the first (superficial, external oblique) and second (intermediate, internal oblique) muscle layers. (**E**) A higher magnification of histology images of hydrogel morphology showing a void surrounded by a lining of cells on day 14 and new tissue growth (black arrows) between hydrogel structures (*) on day 42. Adopted with permission [83].

A polyisocyanide (PIC) hydrogel functionalized with basic fibroblast growth factor, beta-estradiol, and adipose-derived stem cells was evaluated in an abdominal wound healing model in rabbits. The study assessed parameters such as tensile strength, collagen increase, new tissue growth, and immune response. The results showed that the PIC hydrogels with growth factors and stem cells promoted connective tissue healing in rabbit abdominal wounds. This was evidenced by increased tissue stiffness and collagen deposition. This hydrogel system demonstrates potential application in pelvic organ prolapse (POP) surgery to improve surgical outcomes by enhancing connective tissue repair (Figure 5) [83].

A silk fibroin and hyaluronic acid hydrogel crosslinked via Schiff base formation resulted in a pore diameter of approximately 100 μm. The porous structure supported soft tissue regeneration. This SF/HA hydrogel loaded with methylprednisolone demonstrated controlled release of methylprednisolone, compatibility with angiogenesis in ovo, and effective gel formation in vivo in mice. These findings suggest its potential as an effective scaffold for cartilage regeneration, providing valuable insights for treating articular cartilage injuries [84].

A glycopeptide hydrogel containing a tyrosine phosphate group and deferoxamine, which self-assembles and crosslinks through the action of alkaline phosphatase, was evaluated for endothelial cell adhesion, capillary morphogenesis, and in vivo blood capillary generation in mice. The glycopeptide hydrogel formed nanofilament structures that enhanced endothelial cell functions and sustained deferoxamine release, resulting in effective neovascular growth following subcutaneous injection in mice. This suggests its potential use as a scaffold for vascularization in tissue regeneration [85].

A polyvinyl alcohol and silk fibroin hydrogel modified with glycidyl methacrylate and crosslinked using lithium phenyl (2,4,6-trimethylbenzoyl)phosphinate under UV light was analyzed for its mechanical properties, biodegradability, and chondrocyte cell viability for meniscus tissue engineering. The PVA-g-GMA/SF-g-GMA hydrogels exhibited an adjustable compressive modulus, controllable degradation rates, and high chondrocyte viability. These properties make them suitable as biomimetic biphasic injectable hydrogels for meniscus scaffold applications [86].

A hydrogel composed of poly(2-hydroxyethyl methacrylate) (PHEMA), carbon nanofibers (CNFs), and rosette nanotubes (RNTs), assembled in aqueous solution, was studied for its suitability in myocardial applications. This hydrogel was evaluated for cardiomyocyte adhesion and proliferation, electrical conductivity, and mechanical properties. The incorporation of CNFs and RNTs in the hydrogel increased cardiomyocyte density, improved conductivity, and enhanced surface roughness. These enhancements suggest the promising use of this hydrogel for myocardial tissue engineering due to its improved cell functions and material properties [87].

A hydrogel containing stromal-derived factor-1 (SDF-1) and Ac-SDKP peptides was examined for its effects on left ventricle function, angiogenesis, infarct size, and wall thickness improvement in a chronic myocardial infarction model. The study found that the hydrogels with SDF-1 and Ac-SDKP significantly improved cardiac function, enhanced angiogenesis, and reduced infarct size in the chronic MI model. These results demonstrate the synergistic effects of these peptides in promoting cardiac repair and regeneration, indicating their potential for treating chronic heart failure [88].

### Similarities and Differences between Gels Used in Soft Tissue Engineering, Meniscus, and Myocardial Applications

Research on injectable biomimetic gels across soft tissue, meniscus, and myocardial tissue engineering share several methodological similarities. All studies involve the development of hydrogels designed to mimic the natural extracellular matrix to support cell growth, tissue regeneration, and functional recovery. For instance, hydrogels in soft tissue engineering (such as the PIC hydrogel functionalized with growth factors and stem cells) and myocardial applications (like the PHEMA/CNF/RNT composite) both incorporate bioactive molecules to enhance tissue repair and regeneration [83,87]. Additionally, these studies commonly evaluate parameters such as mechanical properties, cell viability, and tissue integration, highlighting the importance of biomechanical compatibility and biological response in the success of these hydrogels [84,86].

Despite the overarching similarities, the methodologies diverge significantly based on the specific tissue applications. In soft tissue engineering, emphasis is placed on enhancing connective tissue repair, as seen with the PIC hydrogel which focuses on tensile strength and collagen deposition [83]. In contrast, meniscus tissue engineering prioritizes mechanical properties such as compressive modulus and degradation rates, essential for supporting the load-bearing function of the meniscus. This is evident in the PVA-g-GMA/SF-g-GMA hydrogel study [86]. Meanwhile, myocardial tissue engineering methodologies often center on electrical conductivity and cardiomyocyte functions, as demonstrated by the PHEMA/CNF/RNT hydrogel, which aims to improve cardiac function through enhanced electrical properties and cell interactions [87].

## 8. Wound Healing and Infection Management

Thise section delves into the development and evaluation of various hydrogels designed for efficient wound healing and infection control. It highlights different types of hydrogels, such as chitosan-based, PEG-based, and gelatin-based, emphasizing their injectability, biodegradability, antibacterial properties, and effectiveness in promoting tissue regeneration. Specific hydrogels, including GC/DP, CQCS@gel, and Cur-hydrogel, among others, are discussed in terms of their composition, crosslinking methods, and in vivo performance, showcasing significant advancements in medical biomaterials aimed at enhancing wound care.

### 8.1. Chitosan-Based Hydrogels

A hydrogel composed of glycol chitosan and dibenzaldehyde-terminated poly(ethylene glycol), crosslinked via Schiff base formation, was investigated for its injectability, biodegradability, and antibacterial properties. This hydrogel, referred to as GC/DP, was tested in vivo using zebrafish embryos for wound healing. The results demonstrated that the GC/DP hydrogel exhibited significant antibacterial activity against Escherichia coli, Pseudomonas aeruginosa, and Staphylococcus aureus. Additionally, it achieved 93.4% wound contraction at 30 days post-wounding, indicating its potential for efficient wound healing with inherent bacteriostatic properties [24].

Formed by crosslinking, a catechol-functionalized quaternized chitosan and dibenzaldehyde-terminated poly(ethylene glycol) hydrogel was developed and named CQCS@gel. This hydrogel was tested for its hemostatic capabilities and effectiveness in healing infected wounds, including the promotion of collagen deposition, hair follicle regeneration, and angiogenesis. The CQCS@gel hydrogel exhibited rapid gelation, strong tissue adhesiveness, contact-active bacterial killing properties, and self-healing and pH-responsive drug release capabilities. It effectively stopped bleeding in acute injuries and promoted the healing of methicillin-resistant Staphylococcus aureus (MRSA)-infected wounds through enhanced collagen deposition and angiogenesis [89].

A nanocomposite hydrogel incorporating graphene oxide, calcium carbonate, and silica into a carrageenan/chitosan matrix was characterized for its blood clotting properties and antibacterial efficacy. The GO/CaCO_3_/SiO_2_ nanocomposite hydrogel demonstrated enhanced blood clotting properties, effective antibacterial activity, and biocompatibility. These features suggest the potential of this hydrogel for addressing hemorrhage and promoting wound healing [90].

A hydrogel composed of phenylazo-terminated Pluronic F127, quaternized chitosan-graft-cyclodextrin, and polydopamine-coated tunicate cellulose nanocrystals was investigated for its self-healing properties, antibacterial activity, drug release capabilities, and wound healing efficacy in a full-thickness skin defect model. This curcumin-loaded hydrogel (Cur-hydrogel) showed responsiveness to light, pH, and temperature for controlled drug release. It also demonstrated inherent antibacterial properties, self-healing ability, and excellent hemostatic performance, effectively promoting wound healing in a full-thickness skin defect model [25].

Recombinant human collagen type III and chitosan hydrogel was studied for its gelation properties, cell proliferation support, antibacterial efficacy, and wound healing capabilities in a full-thickness skin defect model. The rhCol III-CS hydrogel exhibited rapid gelation and complete wound coverage. It facilitated cell proliferation and migration, showed potent antibacterial efficacy, increased collagen deposition, and accelerated full-thickness wound healing, making it a promising multifunctional dressing for skin regeneration [26].

### 8.2. Poly(ethylene glycol) (PEG)-Based Hydrogels

A copolymer hydrogel composed of poly(ethylene glycol) and poly(sulfamethazine ester urethane) (PEG-PSMEU) was developed to form pH- and temperature-sensitive hydrogels. This hydrogel was evaluated for its DNA delivery potential, adhesion to skin, and wound healing capabilities in Sprague-Dawley rats. The PEG-PSMEU copolymer sols transformed into stable gels under body conditions, exhibiting excellent skin adhesion, controlled DNA release over ten days, and accelerated wound healing in rats. It effectively sealed ruptured skin and promoted morphogenesis [27].

A composite hydrogel consisting of poly(ethylene glycol) (PEG) crosslinked with lysozyme amyloid fibrils (LZMF) was assessed for its antibacterial activity, biocompatibility, antiswelling properties, and tissue-adhesive ability both in vitro and in vivo. The PEG-LZMF hydrogel demonstrated superior antibacterial capabilities, inhibition of volume expansion, and good biocompatibility. It also showed strong tissue adhesion, protecting wounds and suppressing pathogen infection through a biomimetic antibacterial mechanism [28].

### 8.3. Gelatin-Based Hydrogels

Dopamine-modified methacrylate gelatin (GMDA) hydrogel, crosslinked with horseradish peroxidase (HRP)/hydrogen peroxide (H_2_O_2_) and ultraviolet (UV) light, was tested for its tissue adhesiveness, antibacterial properties, hemostatic capability, and skin regeneration efficacy in vivo. This GMDA hydrogel demonstrated strong adhesion, tunable elasticity, and superior hemostasis under high blood pressure conditions. Additionally, it exhibited significant antibacterial properties and promoted skin regeneration, especially when combined with epidermal growth factor (EGF) in in vivo studies [91].

An injectable hydrogel adhesive inspired by mussel foot proteins, composed of gelatin modified with thiourea-catechol (Gel-TU-Cat), was evaluated for its tissue adhesion, matrix ductility, and cytocompatibility. This Gel-TU-Cat hydrogel demonstrated rapid curing, strong tissue adhesion, and good cytocompatibility, making it ideal for minimally invasive therapeutic agent delivery and providing local physical barriers for tissue repair [92].

A composite hydrogel made from oxidized sodium alginate and carbohydrazide-modified methacrylated gelatin, embedded with gold/metal-organic framework nanocomposites (Au@ZIF-8), was evaluated for its antibacterial activity and wound healing efficacy under visible light actuation in bacterial and wound models. This composite hydrogel showed enhanced reactive oxygen species (ROS) generation under visible light, remarkable bactericidal activity against Escherichia coli and Staphylococcus aureus, and significantly accelerated wound healing with optimal safety in in vivo studies, effectively combining antibacterial and pro-healing capabilities [93].

### 8.4. Other Polymer-Based Hydrogels

**Figure 6:**
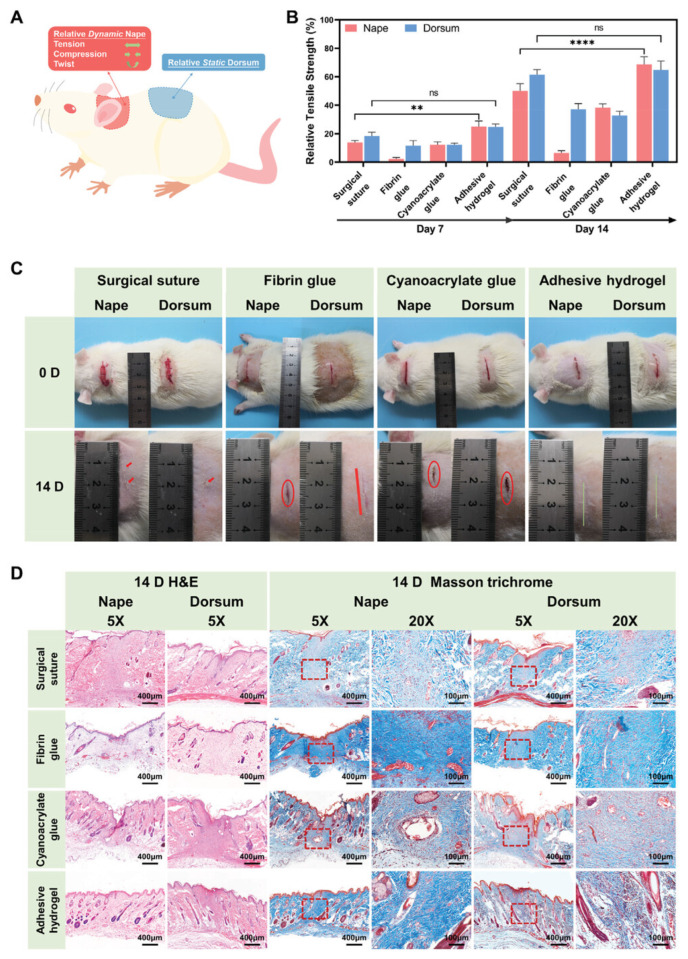
In vivo wound closure and healing performance of the γ-PGA/P-N/CG15 hydrogels in moist and dynamic physiological environment. (**A**) Schematic representation of the full-thickness rat skin incision models that were created on the relatively dynamic nape, in addition to the static dorsum. (**B**) Relative tensile strengths of the healed nape and dorsum skins on the 7th and 14th days post-surgery. All results were normalized to the untreated nape and dorsum skins, respectively. (**C**) Photographs of the full-thickness rat skin incision region on day 0 and the 14th day post-surgery. The red arrows indicate traces of sutures, the red ellipses indicate the unhealed regions of the incisional wounds, and the green lines indicate the healed regions of the incisional wounds. (**D**) H&E and Masson trichrome staining images of the healed skins on the 14th day post-surgery. Values represent the mean and error bars represent the standard deviation (*n* = 5). Adopted with permission [94].

A hydrogel comprising poly(gamma-glutamic acid), amino-functionalized PEGylated poly(glycerol sebacate), and gallic acid-modified chitosan was evaluated for its mechanical resilience, energy dissipation, and wound healing capabilities in a rat skin incision model. The gamma-PGA/PEGS-NH2 hydrogel exhibited strong moist adhesion, high mechanical resilience, and efficient energy dissipation, outperforming fibrin glue in adhesion tests and demonstrating effective wound healing in a full-thickness rat skin incision model (Figure 6) [94].

A G-quadruplex hydrogel composed of guanosine, 4-formylphenylboronic acid, and cytosine-functionalized nucleopeptide was investigated for its antibacterial activity and cytocompatibility in MCF-7 and HEK 293T cell lines. This multicomponent hydrogel exhibited excellent antibacterial activity against a broad range of bacteria and confirmed good in vitro cytocompatibility in both MCF-7 and HEK 293T cell lines, highlighting its promise for preventing localized bacterial infections [95].

Formed from methylacrylyl hydroxypropyl chitosan and Laponite, a self-assembled and photo-crosslinked nanofibrillar hydrogel was tested for its mechanical properties, biocompatibility, and wound healing efficacy in studies involving fibroblast migration and blood vessel formation. The nanocomposite hydrogels exhibited low stiffness, high compressive strength, and anti-swelling properties. They were processed as 3D printable microgels and significantly accelerated wound healing with high biocompatibility and biodegradability [96].

A hydrogel composed of quaternized hydroxyethyl cellulose and mesocellular silica foam (QHM1) was evaluated for its hemostatic properties, antibacterial activities, and wound healing efficacy in a lethal rabbit liver defect model. The QHM1 hydrogel demonstrated instant water-triggered expansion, superabsorbent capacity, reduced plasma clotting time to 59 ± 4% in vitro, and decreased blood loss in the rabbit liver defect model. Additionally, it exhibited remarkable antibacterial activity and promoted wound healing in a full-thickness skin defect model [97].

A multiresponsive injectable hydrogel matrix (MICH) based on catechol-Fe^3+^ coordination, containing vitamin E and extracellular matrix components, was evaluated for its oxidative stress defense, wound integration, and skin regeneration capabilities in burn-wound treatment in vivo. The MICH matrix demonstrated precise scavenging of ROS, reduced tissue ROS production, suppressed inflammation, and enhanced skin regeneration, resulting in low collagen deposition and normal dermal collagen architecture in burn wound healing [98].

Gelatin microspheres (GMs) loaded with platelet-rich plasma (PRP) were investigated for their wound healing rate, vessel density, and inflammation in a rat wound-healing model, utilizing histological and molecular analyses. The GM+PRP formulation demonstrated continuous high release of interleukin-10 and metalloproteinase-3, accelerating wound healing, enhancing angiogenesis, and shortening the healing period compared to PRP alone in the rat wound-healing model [99].

### 8.5. Similarities and Differences between Gels Used in Wound Healing and Infection Management

Various injectable biomimetic gels designed for wound healing and infection management exhibit several common methodological approaches. Primarily, they all focus on incorporating biocompatible and biodegradable materials to enhance wound healing. For instance, both chitosan-based and PEG-based hydrogels utilize natural polymers and their derivatives to promote biocompatibility and facilitate tissue regeneration [24,26,27,28,89]. Another similarity is the emphasis on antibacterial properties; many of these gels incorporate substances with inherent antibacterial activities, such as chitosan and lysozyme, to prevent infections at the wound site [24,25,28,93]. Additionally, rapid gelation and tissue adhesion are key properties frequently evaluated across these studies, ensuring the hydrogels can be easily applied and remain in place during the healing process [89,91,92].

The methods of crosslinking and stabilization vary considerably. Chitosan-based hydrogels often use Schiff base formation or catechol-functionalized quaternized chitosan for crosslinking [24,89], while PEG-based hydrogels might utilize poly(sulfamethazine ester urethane) for its pH- and temperature-sensitive properties [27]. Additionally, the types of additives and functional groups incorporated differ widely, with some gels integrating advanced materials like graphene oxide, calcium carbonate, and silica to enhance mechanical and antibacterial properties [90], and others using gold/metal-organic frameworks for enhanced reactive oxygen species generation and bactericidal activity [93]. Furthermore, the specific in vivo models used for testing also vary, ranging from zebrafish embryos to rat skin defect models, reflecting different focuses on either small or large animal models [24,25,26,27].

## 9. Stem Cell Delivery and Osteoarthritis Treatment

This section discusses various advanced hydrogel-based approaches for stem cell delivery and osteoarthritis treatment. It highlights the development and evaluation of different hydrogels, including GelMA microspheres coated with DMA-MPC polymer, sodium alginate combined with Type I collagen, and amnion membrane with adipose-derived stem cells, among others. These hydrogels have shown promise in improving lubrication, sustaining drug release, reducing inflammation, and promoting tissue regeneration in various animal models. The research underscores the potential of these innovative materials to enhance therapeutic outcomes in osteoarthritis and other regenerative medicine applications.

**Figure 7:**
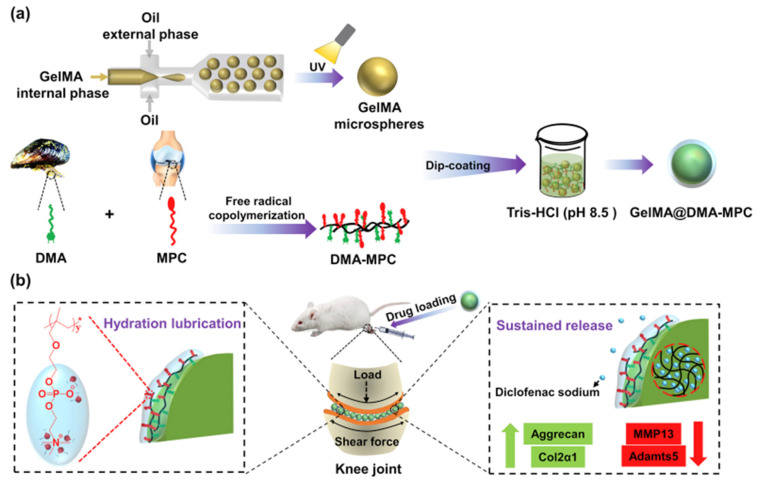
A schematic illustration showing (**a**) the fabrication of GelMA microspheres by microfluidic technology, the synthesis of DMA-MPC polymer by free radical copolymerization, and the design of lubricated GelMA@DMA-MPC microspheres via the dip coating method and (**b**) a treatment option for osteoarthritis by intra-articular injection of the drug-loaded and lubricated GelMA@DMA-MPC microspheres based on the synergistical intervention of enhanced lubrication and sustained drug release. Adopted with permission [100].

Methacrylate gelatin (GelMA) hydrogel microspheres, coated with DMA-MPC (dopamine methacrylamide and 2-methacryloyloxyethyl phosphorylcholine) polymer, synthesized by free radical copolymerization and encapsulating diclofenac sodium (DS), were assessed for their lubrication properties, sustained drug release, and therapeutic effects in a rat osteoarthritis model. These GelMA@DMA-MPC microspheres exhibited enhanced lubrication and sustained drug release, resulting in significant therapeutic effects against osteoarthritis. This included reduced cartilage damage and inflammatory response, as demonstrated by various biological tests and imaging assays (Figure 7) [100].

An injectable hydrogel composed of sodium alginate and Type I collagen was developed to create a better growth microenvironment for mesenchymal stem cells (MSCs), preventing apoptosis and promoting proliferation. The Coll/SA hydrogel displayed a shorter gelation time, biomimetic properties, and significantly improved MSC proliferation and survival. This indicated its potential as a stem cell scaffold for tissue regeneration and organ repair [101].

A hydrogel composed of amnion membrane (AM) combined with adipose-derived stem cells (ADSCs) was evaluated for its potential in reducing inflammation and promoting cartilage regeneration in a collagenase-induced osteoarthritis rat model. The AM hydrogel demonstrated anti-inflammatory and chondroprotective properties, fostering cartilage regeneration. It showed a synergistic effect with ADSCs in reducing inflammation-mediated damage to the articular cartilage in osteoarthritic joints [102].

Hyaluronic acid crosslinked with 1,4-butanediol diglycidyl ether (BDDE) formed hyaluronic acid hydrogels (HAGs), which were evaluated for their viscoelastic properties, swelling behavior, morphology, and rheological properties. These HAGs were tested for cartilage and dentin-pulp complex regeneration. The hydrogels displayed excellent water absorption, pore interconnectivity, and viscoelastic behaviors similar to those of the epidermis, dermis, articular cartilage, and tooth germ. This demonstrated their feasibility as injectable scaffolds for tissue regeneration in a subcutaneous microenvironment [103].

An injectable woven bone-like hydrogel (IWBLH), composed of amorphous calcium phosphate, mineralized collagen fibril, and alginate, was tested in a rat tooth extraction model for alveolar ridge preservation. The IWBLH demonstrated easy handling and effective function in preventing alveolar bone resorption, achieving complete remodeling within four weeks. This indicated its promise as a bone substitute for alveolar ridge preservation [48].

Protein-poly(ethylene glycol) (PEG) hybrid hydrogels (MITCH-PEG), formed from C7 protein and PEG conjugated with proline-rich peptides, were used for the co-delivery of induced pluripotent stem cell-derived endothelial cells (hiPSC-ECs) and growth factors in a mouse hindlimb ischemia model. The MITCH-PEG hydrogels exhibited reversible shear-thinning, self-healing properties, and tunable storage moduli. They effectively co-delivered hiPSC-ECs and VEGF, reduced inflammation, and promoted tissue regeneration, demonstrating protection from cell damage during injection and sustained growth factor release [104].

Pluronic hydrogels, injected as substitutes for the nucleus pulposus, were studied for their stability under fluid flows. In vivo tests in dogs post-discectomy, with a three-month follow-up, indicated that the gels remained present and maintained disc space. Rapid flow rates stabilized the pluronic gels against dissolution, making them viable alternatives for nucleus pulposus replacement. The in vivo tests showed that the gels remained stable and effective in preventing disc space compression in dogs for at least three months [105].

An injectable hydrogel scaffold, derived from decellularized human lipoaspirate and retaining peptides and glycosaminoglycans, was studied for adipose tissue engineering. This hydrogel supported the growth and survival of adipose-derived stem cells (ADSCs) in vitro. The decellularized matrix retained peptides and glycosaminoglycans, self-assembled upon injection, and supported ADSCs, representing a minimally invasive option for adipose tissue engineering and aesthetic improvements [106].

### Similarities and Differences between Gels Used in Stem Cell Delivery and Osteoarthritis Treatment

The methodologies used in injectable biomimetic gels for stem cell delivery and osteoarthritis treatment share several common features. Primarily, the use of natural or synthetic polymers such as methacrylate gelatin (GelMA), sodium alginate, Type I collagen, and hyaluronic acid is prevalent. These materials provide a conducive environment for cell encapsulation and proliferation due to their biocompatibility and biomimetic properties [100,101,102,103]. Moreover, many studies emphasize the importance of hydrogel physical properties, such as gelation time, viscoelasticity, and pore interconnectivity, which are critical for mimicking the extracellular matrix and supporting tissue regeneration [103,104]. Another commonality is the incorporation of bioactive agents or drugs, such as diclofenac sodium or growth factors, which aid in reducing inflammation and promoting tissue repair [100,104]. Lastly, most methodologies involve in vivo testing in animal models to evaluate the therapeutic efficacy and biocompatibility of the hydrogels [100,102,105].

The specific approaches and materials used for these hydrogels, despite their similarities, have been shown to be different. For example, GelMA microspheres coated with DMA-MPC polymer were specifically designed for enhanced lubrication and sustained drug release in osteoarthritis treatment, highlighting their unique application in joint therapy [100]. In contrast, sodium alginate and Type I collagen hydrogels focused on creating a microenvironment that supports mesenchymal stem cell proliferation and survival for broader tissue regeneration applications [101]. The use of amnion membrane combined with adipose-derived stem cells represents another distinct approach aimed at reducing inflammation and promoting cartilage regeneration, which differs significantly from the drug-focused methodology of GelMA microspheres [102]. Additionally, the hydrogel composed of hyaluronic acid crosslinked with BDDE was specifically tested for both cartilage and dentin-pulp complex regeneration, showcasing its versatile application in different tissue types [103]. Another unique example is the injectable woven bone-like hydrogel (IWBLH) designed for alveolar ridge preservation, focusing on preventing bone resorption post-tooth extraction [48].

## 10. Neural Tissue Engineering

Hydrogels in neural tissue engineering, based on materials like gelatin, collagen, and synthetic polymers, demonstrate enhanced electrical conductivity, improved mechanical properties, and excellent biocompatibility. They support neural cell growth and differentiation, making them promising candidates for minimally invasive neurological therapies, neural tissue repair, and regenerative medicine applications.

Electroconductive injectable hydrogels based on gelatin and poly(3,4-ethylenedioxythiophene)-poly(styrenesulfonate) (PEDOT) have been evaluated for their potential in neural tissue regeneration. These hydrogels have demonstrated enhanced electrical conductivity, improved shear modulus, reduced gelation time, and decreased swelling ability. Additionally, they have been shown to support the growth of primary rat cortical astrocytes. The tunable properties of these hydrogels make them promising candidates for minimally invasive neurological therapies and brain tissue repair [107].

Hydrogels made of collagen, laminin, hyaluronic acid, and chondroitin sulfate proteoglycan, without the use of external crosslinking agents, have been assessed for their effectiveness in neural stem cell (NSC) delivery. These hydrogels exhibited excellent injectability, maintained gel integrity, and promoted the survival and differentiation of NSCs. The cellular binding to the hydrogels through surface integrins facilitated neurite outgrowth, indicating their utility in neural tissue engineering and regenerative medicine. These properties suggest that such hydrogels can be effectively used for neural tissue repair and the delivery of stem cells [108].

Injectable hydrogels composed of various biodegradable polymers that mimic the extracellular matrix (ECM) have been evaluated for central nervous system (CNS) tissue engineering. These hydrogels provide a three-dimensional scaffold that supports CNS regeneration by mimicking the properties of the ECM, promoting extracellular matrix remodeling, and facilitating neural tissue regeneration with minimal invasiveness. This approach highlights the potential of these hydrogels for CNS repair and regeneration [109].

Glycosaminoglycan analogues based on sulphonate-containing polymers were developed for nucleus pulposus repair. These hydrogels were tested for their fixed charge density, hydration, and osmotic responsiveness in vitro, and underwent preliminary biomechanical tests in a degenerate explant model. The hydrogels exhibited appropriate fixed charge density, hydration, and osmotic responsiveness, restoring stiffness and function in degenerate disc models by mimicking the role of glycosaminoglycans in vivo [110].

### Similarities and Differences between Gels Used in Neural Tissue Engineering

They all aim to mimic the extracellular matrix (ECM) to support neural cell growth, differentiation, and tissue regeneration. These gels exhibit excellent injectability, biocompatibility, and mechanical properties suitable for minimally invasive therapies [107,108,109]. Additionally, they promote cellular interactions through surface integrins, enhancing neurite outgrowth and tissue repair. However, there are notable differences among these hydrogels. The electroconductive hydrogels based on gelatin and PEDOT focus on electrical conductivity and reduced gelation time [107], while the hydrogels made of collagen, laminin, hyaluronic acid, and chondroitin sulfate proteoglycan prioritize injectability and NSC delivery without external crosslinking agents [108]. In contrast, the biodegradable polymer-based hydrogels emphasize mimicking ECM properties and promoting extracellular matrix remodeling for CNS regeneration [109].

## 11. Specialized Applications

This section discusses various specialized applications of injectable hydrogel systems in medical treatments and tissue engineering. It highlights the development and evaluation of different hydrogel formulations designed to mimic the extracellular matrix (ECM), support cell growth, and promote tissue regeneration. These hydrogels are engineered for specific medical applications, such as retinal neovascularization, mandibular osteomyelitis, cartilage repair, and cardiac tissue engineering. The studies emphasize the importance of tunable physical and biochemical properties to create optimal environments for cell proliferation and differentiation, showcasing the potential of these hydrogels in minimally invasive treatments.

An injectable hydrogel system using aminated hyaluronic acid and aldehyde-functionalized Pluronic 127 has been evaluated for its potential in treating retinal neovascularization (RNV). This system, named HP@Ran, demonstrated excellent injectability, self-healing properties, and sustained release of ranibizumab for over seven weeks. The results showed a significant reduction in vascular leakage and neovascularization in a rabbit RNV model, indicating its potential for effective RNV treatment [111].

Injectable gentamicin-collagen hydrogels (GNT-COLL) and nanohydroxyapatite-loaded hydrogels (GNT-COLL/nHA) were tested in a rabbit model of mandibular osteomyelitis for local treatment efficacy, bone preservation, and infection suppression. The results indicated that these hydrogels were more effective in suppressing osteomyelitis and preserving bone than systemic gentamicin, with no recurrence for up to 12 weeks. This supports the use of composite hydrogels for the treatment of osteomyelitis [112].

Bioinspired injectable hydrogels made from Bombyx mori silk fibroin, carboxymethyl cellulose, and gelatin were designed for cartilage tissue engineering. These hydrogels facilitated dynamic stiffening and contraction, mimicking the natural development of cartilage. They promoted chondrogenesis and demonstrated potential for cartilage repair, highlighting their application in regenerative medicine [113].

Blended hydrogels composed of mulberry silk (Bombyx mori) and non-mulberry silk (Antheraea assama), loaded with interleukin-4 and dexamethasone, were evaluated for their impact on islet viability, insulin secretion, endothelial cell maintenance, and M2 macrophage polarization. These hydrogels supported sustained release of IL-4 and dexamethasone, promoting M2 macrophage polarization and preserving islet physiology, showing promise for type 1 diabetes treatment [114].

Injectable anisotropic nanocomposite hydrogels, based on hydrazone cross-linked poly(oligoethylene glycol methacrylate) and magnetically aligned cellulose nanocrystals (CNCs), promoted the differentiation of skeletal muscle myoblasts into oriented myotubes. The CNC alignment during gelation was maintained, providing mechanical properties and directing myotube alignment, representing a significant advancement in anisotropic biomimetic scaffolds for muscle tissue engineering [115].

An injectable, near-infrared (NIR) and pH-responsive nanocomposite hydrogel using sodium alginate-graft-dopamine and polydopamine-Fe(III)-doxorubicin nanoparticles was developed for melanoma treatment and skin regeneration. This hydrogel provided photothermal therapy, chemotherapy, and nanozyme synergetic therapy. It delivered anti-cancer agents precisely, converted light into heat to kill cancer cells, and released doxorubicin continuously, promoting skin regeneration by scavenging reactive oxygen species and killing bacteria [116].

Needle-injectable cryogel scaffolds, hybridized with calcium peroxide microparticles to produce hydrogen peroxide, were synthesized using polyvinyl alcohol and gelatin. These microcomposite cryogels exhibited antimicrobial properties against MRSA and Pseudomonas aeruginosa, showed negligible cytotoxicity toward murine fibroblasts, and prevented activation of dendritic cells. They were evaluated for tissue integration, biodegradation, and minimal host inflammatory response in vivo, indicating their potential for antimicrobial applications and tissue repair [117].

A dual-crosslinked hydrogel system based on chitosan functionalized with methacryloyl and tricine moieties was found to be processable at physiological pH and was assessed for its cytocompatibility with MC3T3-E1 pre-osteoblasts. The CHTMA-tricine hydrogels demonstrated significant toughness, cell viability, and potential for 3D bioprinting applications. This hydrogel system promises advancements in tissue engineering and regenerative medicine [118].

The MAX8B injectable peptide hydrogel system was developed for bioengineering a three-dimensional trabecular meshwork scaffold. This scaffold was assessed for its mechanical and bio-instructive properties, shear-thinning ability, and biocompatibility for human trabecular meshwork (hTM) growth. The engineered scaffold facilitated proper hTM growth and proliferation, and mimicked the physiological response to dexamethasone treatment, suggesting its potential as an injectable trabecular meshwork implant [119].

Peptide-polyurea hybrids, using poly(epsilon-carbobenzyloxy-L-lysine)-b-PEG-b-poly(epsilon-carbobenzyloxy-L-lysine) and poly(beta-benzyl-L-aspartate)-b-PEG-b-poly(beta-benzyl-L-aspartate), were examined for their hydrogelation, microstructure, rheological properties, and injection recovery. These hybrids exhibited solid-like properties and thermal stability, with the peptide segment length dictating gel strength and resistance to deformation. The hydrogels showed unique softening transitions at specific temperatures, indicating their potential for various biomedical applications [120].

A disc-derived extracellular matrix (ECM) injectable hydrogel functionalized with chondroitin sulfate was evaluated for intervertebral disc regeneration. The hydrogel promoted matrix deposition by nasal chondrocytes, increasing sGAG production and the synthesis of collagen type II. The inclusion of chondroitin sulfate enhanced sGAG production and collagen type II synthesis, promoting nucleus pulposus-like matrix deposition by nasal chondrocytes, and demonstrating potential for intervertebral disc regeneration [121].

Poly(l-glutamic acid)-based injectable hydrogel decorated with RGD was developed for tunable bioactivity and cell adhesion. This hydrogel was evaluated for cell-matrix interaction, cell adhesion, and proliferation, with controllable RGD density via disulfide bonds for dynamic regulation of cell behaviors. The RGD conjugation allowed precise control over hydrogel bioactivity, providing a strategy to develop ECM-mimicking scaffolds for dynamically regulating cell adhesion [122].

An injectable reverse thermal gel (RTG) was developed for minimally invasive coverage of myelomeningocele (MMC) defects. This gel was tested in vitro for stability in amniotic fluid and in vivo in a mouse MMC model for defect coverage and inflammation response. The RTG maintained stability in amniotic fluid for six months and successfully formed a stable gel over MMC defects in mice, demonstrating more than 50% coverage and no inflammation. This indicates its potential for minimally invasive prenatal MMC repair [123].

A reverse thermal injectable gel functionalized with carbon nanotubes (RTG-CNT) was developed for cardiac tissue engineering. This system promoted cardiomyocyte survival, alignment, proliferation, and function, transitioning from a solution at room temperature to a gel at body temperature. The RTG-CNT system supports long-term cardiomyocyte survival, promotes alignment and proliferation, and improves cardiac function, representing a minimally invasive tool for cardiac tissue engineering [50].

### Similarities and Differences between Gels Used in Specialized Tissue Engineering Applications

All studies focused on developing hydrogels that mimic the extracellular matrix (ECM) to support cell growth and tissue regeneration. These hydrogels are designed to be injectable, providing minimally invasive treatment options that enhance patient recovery and reduce surgical risks [107,108,109,115,119,123]. These formulations emphasize the importance of tunable physical and biochemical properties, such as gelation time, mechanical strength, and bioactivity, to create an optimal environment for cell proliferation and differentiation [107,111,113,116,118]. Many of these studies highlighted the use of natural polymers like gelatin, hyaluronic acid, and collagen, which are known for their biocompatibility and ability to support cellular functions [107,108,110,113,117].

The types of polymers and crosslinking mechanisms vary widely. Some studies use synthetic polymers like poly(3,4-ethylenedioxythiophene) (PEDOT) for enhanced electrical conductivity [107], while others use natural polymers such as silk fibroin for dynamic stiffening and contraction [113]. The intended applications also differ significantly, ranging from neural tissue engineering [107,108,109] to specialized applications like retinal neovascularization treatment [111], mandibular osteomyelitis [112], and melanoma treatment [116]. Furthermore, the specific functional additives and their roles differ, as some studies incorporate growth factors or drugs for sustained release and targeted therapy [111,112], while others focus on mechanical properties and cell alignment using components like magnetically aligned cellulose nanocrystals [115] or carbon nanotubes [50].

## 12. Innovative Drug Delivery Systems

This section explores innovative drug delivery systems designed to improve therapeutic efficacy and reduce toxicity. These systems include hydrogels and nanoparticles that enhance drug solubility, stability, and controlled release. They demonstrate potential for localized and sustained cancer treatments, incorporating features like self-healing, injectability, and responsiveness to environmental triggers, thus offering advanced solutions for targeted therapy and regenerative medicine applications.

A multi-level drug release platform incorporating curcumin-loaded liposomes, chitooligosaccharides, phospholipids, and a thiolated chitosan hydrogel was evaluated for its water solubility, encapsulation efficiency, stability, cellular intake, and bioactivity. This platform significantly improved the solubility, encapsulation efficiency, and stability of curcumin, enhancing its inhibitory effect on MCF-7 cells compared to conventional formulations. The hydrogel component facilitated local immobilization and sustained release of curcumin, demonstrating its potential for more effective localized cancer treatment [124].

A hydrogel comprising reactive oxygen species (ROS)-sensitive tegafur (TF)-protoporphyrin IX (PpIX) heterodimers encapsulated in temperature-sensitive chitosan and silk sericin was evaluated for its synergistic chemotherapy and photodynamic therapy potential in breast cancer. The hydrogel enabled “on-demand” drug release when exposed to 630 nm laser irradiation, enhancing drug effectiveness while reducing toxicity. In both in vivo and in vitro studies, the hydrogel significantly increased ROS production, controlled drug release, and improved tumor-cell-killing capabilities, demonstrating substantial improvements in breast cancer treatment outcomes [125].

Self-healable, injectable eutectogel based on deep eutectic solvents and poly(vinyl alcohol) was assessed for its hydrogen bonding interactions, morphology, mechanical strength, thixotropic behavior, phase transition, and drug delivery potential. This multifunctional eutectogel exhibited self-healing, adhesive, and moldable properties along with high drug absorptivity and stability. It efficiently delivered the anticancer drugs 5-fluorouracil and curcumin, showing promising chemotherapeutic activity against MCF-07 human breast cancer cell lines [126].

A hyaluronic acid-based organo-hydrogel influenced by click chemistry was evaluated for its biocompatibility, eco-friendliness, self-healing properties, and pharmaceutical potential. This biomimetic, injectable hydrogel demonstrated excellent biocompatibility and eco-friendliness, making it suitable for various low-toxicity therapeutic applications. The incorporation of click chemistry highlighted its importance in enhancing pharmaceutical formulations and bioavailability [127].

Hydrogels based on paclitaxel-loaded bovine serum albumin (BSA) nanoparticles cross-linked with o-phthalaldehyde-terminated 4-armed poly(ethylene glycol) (4aPEG-OPA) were evaluated for sustained drug release, tumor adhesion, and inhibition of colon and breast cancer cells. When injected peritumorally in mice, these nanocomposite hydrogels showed enhanced antitumor efficacy and prolonged survival with low systemic toxicity. The hydrogels sustained paclitaxel release over 30 days, demonstrated notable cancer cell inhibition, and adhered firmly to tissues, thus enhancing localized tumor therapy in animal models [128].

A firefly luciferin-inspired hydrogel with redox-triggering capability, using protected macromers, was evaluated for gelation onset, kinetics, and cytocompatibility. This system enabled precise control over gelation, making it suitable as an injectable cell-encapsulating hydrogel. The redox-triggerable nature of this hydrogel allowed fine-tuning of gelation onset and kinetics, expanding its application range for 3D cell culture and other biomedical uses, and demonstrating its potential for advanced therapeutic and regenerative medicine applications [129].

### Similarities and Differences between Gels Used in Innovative Drug Delivery Systems

All of these hydrogel systems aim to improve drug solubility, stability, and controlled release, enhancing therapeutic efficacy and reducing toxicity. They also demonstrate advanced properties like injectability, biocompatibility, and responsiveness to environmental triggers, making them suitable for localized and sustained treatment applications [124,125,126,127,128,129]. On the other hand, the multi-level drug release platform focuses on enhanced solubility and sustained release of curcumin for localized cancer treatment [124], while the ROS-sensitive tegafur-protoporphyrin IX heterodimers target synergistic chemotherapy and photodynamic therapy with temperature-sensitive release [125]. The self-healable eutectogel highlights its moldable and adhesive properties for efficient drug delivery [126], whereas the hyaluronic acid-based organo-hydrogel emphasizes eco-friendliness and low-toxicity therapeutic applications [127]. The paclitaxel-loaded BSA nanoparticles prioritize sustained drug release and tumor adhesion for prolonged cancer treatment [128], and the firefly luciferin-inspired hydrogel focuses on precise control over gelation for 3D cell culture and regenerative medicine [129].

## 13. Miscellaneous Applications

This section explores various advanced hydrogel formulations and their diverse biomedical applications. It discusses how different types of hydrogels, including GelMA/SerMA, hyaluronic acid, polyisocyanopeptide, and others, are designed and evaluated for specific medical purposes. Applications range from endometrial regeneration, controlled drug delivery, T cell therapy, diabetes treatment, neural regeneration, to tissue engineering. Each hydrogel’s unique properties, such as self-healing, injectability, biocompatibility, and targeted delivery, highlight their potential in improving therapeutic outcomes and advancing regenerative medicine.

A methacrylate gelatin (GelMA) and methacrylate sericin (SerMA) hydrogel loaded with human umbilical cord mesenchymal stem cells (HUMSC) was evaluated for its potential in endometrial regeneration and fertility restoration. In vivo experiments demonstrated that this hydrogel significantly increased endometrial thickness, improved interstitial fibrosis, and enhanced embryo transfer receptivity. These findings indicate that the GelMA/SerMA@HUMSC hydrogel has significant potential for repairing or regenerating damaged endometrium, thereby improving fertility outcomes [130].

A hyaluronic acid hydrogel modified by adamantane and cyclodextrin through a proteolytically degradable peptide tether was assessed for its formation, proteolytic degradation, shear-thinning, and self-healing properties, and potential for in vivo subcutaneous injection. The hydrogel demonstrated shear-thinning and self-healing abilities, with selective degradation by collagenases or MMP-2. These properties facilitated therapeutic delivery and bioresponsive degradation both in vitro and in vivo, highlighting its potential for controlled drug delivery applications [131].

Polyisocyanopeptide hydrogels with azide-terminated monomers were explored for their potential in T cell expansion and delivery. In vitro T cell culture and in vivo subcutaneous injection studies revealed that these hydrogels enhanced cell survival, expansion, and migration without inducing inflammation. These findings indicate that PIC hydrogels could improve adoptive T cell therapy strategies by facilitating T cell survival and expansion in a non-immunogenic manner [132].

A poly(N-isopropylacrylamide-co-dextran-maleic acid-co-3-acrylamidophenylboronic acid) hydrogel was evaluated for its glucose and thermo-responsive behavior, biocompatibility, and ability to regulate blood glucose levels in real-time. This hydrogel demonstrated glucose-dependent insulin release and successfully restored blood glucose levels in diabetic rats without causing an inflammatory response. These results present a promising strategy for diabetes treatment through real-time glycaemic regulation [133].

A hydrogel grafted with dihydroxyphenylalanine on chitosan and incorporating designer peptides was developed for neural regeneration following spinal cord injury. It was evaluated for its injectability, self-healing capabilities, tissue adhesion, and functional recovery outcomes. The hydrogel significantly enhanced motor and sensory function recovery, bladder repair, and neural regeneration by promoting synapse formation and myelin regeneration, showing substantial promise for spinal cord injury repair and other tissue regeneration applications [134].

Injectable, pore-forming double-network hydrogels, fabricated via stepwise gelation and phase separation, were evaluated for their permeability, toughness, cell encapsulation, and delivery capabilities. These hydrogels maintained their integrity under prolonged high-frequency biomechanical stimulations, making them suitable for dynamic tissue repair. They also supported cell proliferation and spreading, indicating their potential for various tissue engineering and biomedical applications [135].

Co-spheroids composed of neural stem cells (NSCs) and endothelial cells (ECs), encapsulated in a gelatin-based hydrogel, were assessed for their angiogenic potential and neurovascular network formation capabilities. The encapsulation in gelatin-based hydrogel enhanced the viability of the cells and promoted the formation of tube-like structures, indicative of angiogenesis and neurovascular network formation. These results suggest that NSC/EC co-spheroids in a gelatin-based hydrogel could be used to build biomimetic neurovascular constructs for neuroregeneration [136].

### Similarities and Differences between Gels Used Drug Delivery Applications

Many methodologies used in developing injectable biomimetic gels for drug delivery share common features, including the incorporation of hydrogels to facilitate drug encapsulation and sustained release. Thiolated chitosan hydrogel was utilized to improve the solubility, encapsulation efficiency, and stability of curcumin, enhancing its therapeutic effects against MCF-7 cells [124]. Similarly, a temperature-sensitive chitosan and silk sericin hydrogel enabled controlled release and synergistic chemotherapy and photodynamic therapy for breast cancer [125]. These hydrogels also frequently exhibit self-healing properties, as demonstrated by a eutectogel based on deep eutectic solvents and polyvinyl alcohol that efficiently delivered anticancer drugs while maintaining structural integrity [126]. Furthermore, the use of natural polymers like hyaluronic acid and chitosan is a recurring theme, providing biocompatibility and eco-friendliness essential for biomedical applications [127].

Despite many commonalities, there are distinct differences in the specific design and application of these hydrogels. The incorporation of ROS-sensitive tegafur-protoporphyrin IX heterodimers in a chitosan and silk sericin hydrogel highlights a unique approach leveraging laser irradiation to trigger drug release, enhancing chemotherapy and photodynamic therapy outcomes [125]. In contrast, a methacrylate gelatin and sericin hydrogel was designed to support stem cell delivery for endometrial regeneration, demonstrating improvements in endometrial thickness and fertility restoration [130]. Additionally, a polyisocyanopeptide hydrogel tailored for T cell expansion and delivery demonstrated enhanced cell survival and migration without inducing inflammation, showcasing its potential for adoptive T cell therapy [132]. These variations reflect the diverse therapeutic targets and functional requirements driving the customization of hydrogel formulations.

## 14. Key Factors in Selecting an Injectable Biomimetic Gel for a Specific Application

Selecting an injectable biomimetic gel for tissue engineering and regenerative medicine involves several critical factors. These considerations ensure the gel’s effectiveness and safety across diverse applications, ranging from bone and cartilage repair to soft tissue regeneration and wound healing. The key factors include biocompatibility, mechanical properties, biodegradability, bioactive components, injectability, and practical considerations related to clinical use and production.

Biocompatibility is the foremost consideration when choosing an injectable biomimetic gel. The gel must support cell viability and proliferation without triggering an adverse immune response. Ensuring biocompatibility allows the gel to integrate seamlessly with the host tissue, whether it is for bone, cartilage, soft tissue, or wound healing. Hyaluronic acid-based hydrogels, for instance, are renowned for their excellent biocompatibility and low toxicity, making them suitable for a variety of therapeutic applications [127].

The mechanical properties of the gel, including compressive strength, elasticity, viscoelasticity, and gelation time, must be tailored to match the requirements of the target tissue. For bone tissue engineering, the gel needs to have adequate compressive strength and elasticity to support the biomechanical environment of the bone [21,36]. For cartilage or myocardial tissue engineering, the gel must offer sufficient mechanical stability to support chondrocytes or cardiomyocytes while facilitating natural tissue functions [41,87]. Additionally, properties such as self-healing and maintaining structural integrity under physiological conditions are particularly important for dynamic tissues [34,126].

The rate of biodegradation of the gel must align with the rate of new tissue formation. This balance ensures that the gel provides temporary support and degrades as the new tissue forms, preventing premature degradation that could compromise tissue regeneration or excessive persistence that might interfere with the natural healing process [65,107,108]. Gels must be designed to degrade safely without eliciting adverse reactions, which is crucial for applications like wound healing and infection management [24,26,27,28].

Incorporating bioactive components into the gel can significantly enhance its therapeutic potential. For bone tissue engineering, components like hydroxyapatite, BMPs, or growth factors improve osteoinductive and osteoconductive properties [19,56]. Similarly, in cartilage tissue engineering, bioactive molecules such as kartogenin or factors like anhydroicaritin in chitosan-based hydrogels can promote chondrocyte proliferation and matrix production [67,80]. The ability to deliver bioactive molecules, such as growth factors and drugs, is also essential for promoting tissue regeneration and healing across various applications [111,114,116].

The injectability and ease of use of gels are crucial for clinical applications, particularly for minimally invasive procedures. Gels should be easy to handle, form rapidly in situ, and provide a stable scaffold for tissue regeneration [10,55,111,119,123]. This is important across various applications, from bone and cartilage repair to soft tissue engineering and wound healing [70,72,89,91,92,137].

Practical considerations for clinical use include the gel’s potential for large-scale production and consistency in quality. Ensuring scalability and quality consistency is vital for translating these biomaterials from the laboratory to clinical settings, addressing challenges such as variability in stem cell quality, and ensuring consistent therapeutic outcomes [105,106]. Additionally, the gel’s ability to encapsulate and sustain the release of therapeutic agents or stem cells directly impacts its effectiveness in promoting tissue regeneration [100,104].

While the above factors are generally applicable, specific applications may require additional considerations. For instance, in neural tissue engineering, the gel must offer electrical conductivity and dynamic stiffening properties to support neural functions [107,113]. For diabetic treatments, glucose-responsive hydrogels designed for real-time blood glucose regulation have shown promise [133]. In neurovascular applications, co-spheroids of neural stem cells and endothelial cells encapsulated in a gelatin-based hydrogel illustrate the potential for tailored regenerative medicine solutions [136]. Furthermore, self-healing hydrogels demonstrate significant potential in wound management, offering accelerated wound closure, tissue regeneration, and adaptable mechanical properties suitable for diverse clinical applications [34].

## 15. Tests and Assessments

When developing an injectable biomimetic gel, various tests need to be conducted to ensure its efficacy, safety, and functionality. These tests can be broadly categorized into physical and chemical properties, in vitro, in vivo, and in silico assessments. Below is is an overview outlining the essential tests under each category.

### 15.1. Physical and Chemical Properties

**Injectability:** To evaluate the ease with which the gel can be injected through a syringe, tests should measure the force required to expel the gel at different temperatures and flow rates. This includes rheological assessments to determine viscosity and flow behavior [1,2,8,12,36,37,53,54,55,59,61].

**Mechanical Properties** Assessments of the gel’s mechanical properties are crucial. These include tests for strength, flexibility, extensibility, and toughness. Methods such as tensile testing, compression testing, and cyclic loading can provide insights into the gel’s mechanical robustness and durability [1,3,4,5,8,9,12,14,18,19,21,36,37,38,39,43,45,51,52,53,54,57,58,59,60,61,62,63].

### 15.2. Self-Healing and Adhesive Properties

**Self-Healing:** The self-healing capability of the gel can be evaluated by making incisions or punctures and observing the time and effectiveness of the gel’s healing process. Techniques such as optical microscopy and mechanical testing can be employed to monitor and quantify the healing efficiency [1,2,4,5,14,39,45,52,53,54,55].

**Adhesive Properties:** The adhesive strength of the gel to various biological and synthetic surfaces can be tested using lap shear and peel tests. These tests determine the gel’s ability to adhere and maintain contact under different conditions [2,3,4,5,8,14,40,52].

### 15.3. Biocompatibility and Cytocompatibility

**Biocompatibility:** Biocompatibility tests ensure that the gel does not induce any adverse immune responses when introduced to biological systems. These tests involve the use of animal models and human cells to evaluate inflammatory responses, tissue integration, and overall biocompatibility [2,5,12,14,15,21,38,41,42,43,45,54,68,69,82].

**Cytocompatibility:** Cytocompatibility tests assess the gel’s compatibility with various cell types, ensuring that it supports cell viability and proliferation. This can be evaluated using assays like MTT, live/dead staining, and flow cytometry [3,4,39,53,55,75,81,82].

### 15.4. Wound Healing and Hemostasis

**Wound Healing Promotion:** Tests to determine the gel’s ability to promote wound healing involve both in vitro scratch assays and in vivo wound models. These tests measure the rate of wound closure, re-epithelialization, and collagen deposition [1,2,17,24,51,83,93].

**Hemostasis and Antihemorrhagic Properties:** To evaluate the gel’s hemostatic properties, tests such as coagulation assays and bleeding time measurements in animal models are conducted. These tests measure the gel’s ability to stop bleeding and facilitate clot formation [1,2,4,89].

### 15.5. Angiogenesis and Osteogenesis

**Angiogenesis:** The ability of the gel to promote new blood vessel formation is assessed using in vitro tube formation assays and in vivo models of revascularization. These tests measure the extent and functionality of new blood vessels formed in response to the gel [17,20,51,56,65].

**Osteogenesis:** Osteogenesis tests determine the gel’s capability to support bone formation and mineralization. In vitro tests with osteoblasts and in vivo bone defect models are used to evaluate bone integration and the formation of mineralized tissue [9,14,15,19,20,21,23,36,43,46,56,57,60,61,62].

### 15.6. Cellular Interaction and Behavior

**Cell Adhesion and Proliferation:** Tests to measure cell adhesion and proliferation involve culturing various cell types on the gel and using assays like cell counting, proliferation markers, and imaging techniques to assess how well cells adhere to and proliferate on the gel surface [3,9,12,14,15,17,21,35,36,37,38,39,42,43,45,53,54,55,57,58,59,60,61,62,63,64].

**Stem Cell Encapsulation and Culture:** These tests evaluate the gel’s ability to encapsulate and support the growth and differentiation of stem cells. Techniques such as encapsulation efficiency, viability assays, and differentiation markers are used [9,12,37,45,52,59,61].

### 15.7. Antibacterial and Antioxidant Properties

**Antibacterial Activity:** The antibacterial properties of the gel can be assessed using standard microbiological assays against common pathogens. This includes measuring the zone of inhibition, bacterial adhesion, and growth kinetics [1,2,63].

**Antioxidant Properties:** Antioxidant assays evaluate the gel’s ability to neutralize reactive oxygen species (ROS). Methods such as DPPH and ABTS assays can be used to quantify the gel’s antioxidant capacity [14].

### 15.8. Drug Delivery and Therapeutic Effects

**Sustained Drug Release:** Tests for sustained drug release involve loading the gel with a therapeutic agent and measuring the release profile over time using methods like HPLC and UV spectrophotometry. These tests ensure controlled and prolonged release of the drug [13,47].

**Therapeutic Effects:** Evaluating therapeutic effects involves testing the gel in relevant disease models, such as tumor ablation or growth suppression in cancer models. This includes assessing the gel’s efficacy in delivering therapeutic agents and its impact on disease progression [47,125].

### 15.9. In Vivo and In Vitro Testing

**In Vivo Testing:** In vivo tests involve the application of the gel in animal models to assess biocompatibility, biodegradability, and therapeutic efficacy. Parameters such as immune response, tissue integration, and functional outcomes are measured [1,2,3,9,13,14,15,17,19,20,21,35,36,37,43,45,46,47,51,55,56,57,58,59,60,61,62,63,64].

**In Vitro Testing:** In vitro testing involves a series of cell culture assays to evaluate cell viability, proliferation, differentiation, and function in the presence of the gel. These tests provide preliminary data on biocompatibility and biological performance [1,9,15,18,21,35,37,38,39,42,43,46,47,51,52,54,55,56,57,58,59,60,62,63,64].

Conducting these comprehensive tests will provide a robust evaluation of the injectable biomimetic gel’s properties and potential applications, ensuring its safety and efficacy for clinical use.

## 16. Overall Outcomes

Injectable biomimetic gels have shown substantial progress across various biomedical fields due to their enhanced biocompatibility, multifunctional properties, and efficacy in controlled delivery systems. These hydrogels integrate well with tissues and demonstrate a variety of beneficial functionalities, including improved wound healing, antihemorrhagic properties, and support for cellular activities such as proliferation and differentiation. Specific examples include ECM-mimetic hydrogels that promote tissue integration post-injection [12] and thermoresponsive hydrogels aiding bone formation [43].

The multifunctional nature of these hydrogels also includes self-healing capabilities and thixotropic properties that support tissue regeneration, such as self-healing bioinspired hydrogels for wound closure [2] and silk-hydroxyapatite hydrogels that promote osteogenesis [46]. Furthermore, bifunctional hydrogels with photothermal effects are being explored for applications in tumor therapy and bone regeneration [47].

Controlled delivery systems in hydrogels are another area of significant advancement. For instance, hypoxia preconditioned serum-fibrin hydrogels enhance angiogenesis by delivering growth factors [51], and alginate bioconjugate hydrogels sustain BMP-2 release for bone engineering [13]. Hydrogels like these offer targeted delivery that enhances tissue regeneration and repair, including silk fibroin/hyaluronic acid hydrogels for cartilage repair [84].

In broader tissue regeneration, a variety of hydrogels are being utilized to support vascularization, chondrogenesis, and bone regeneration. Examples include MSC-laden GelMA-HAP-SN hydrogels that excel in bone regeneration [9] and myocardial hydrogels that improve heart function and angiogenesis [88]. Injectable hydrogels are also being used for neural tissue engineering and osteoarthritis treatments, where they promote neural stem cell survival [108] and reduce inflammation [102].

Hydrogels with specialized applications, such as electroconductive hydrogels for neural tissue regeneration [107] and hydrogels for retinal neovascularization treatment [111], illustrate the versatility and potential of these materials in various therapeutic areas. Multi-level drug release platforms and biomimetic eutectogels are further extending the capabilities of hydrogels in delivering therapeutic agents effectively [124,126].

## 17. Limitations

Despite these advancements, hydrogels face several limitations that need to be addressed for broader clinical application. One significant challenge is enhancing the mechanical resilience of hydrogels to withstand physiological stresses. For example, dopamine-modified PEG nanocomposite hydrogels require improved long-term stability and strength [3], and calcium phosphate cement-based hydrogels need optimization for osteoporotic fractures [23].

Another critical issue is aligning the degradation rates of hydrogels with tissue regeneration timelines. Mussel-inspired injectable hydrogels, for example, need precise degradation control to be effective [5]. Similarly, hydrogels designed for bone regeneration must have carefully controlled degradation rates to match the regeneration process [65].

The complexity of hydrogel synthesis also presents a barrier to large-scale production and clinical use. Dual crosslinking mechanisms and complex formulations, such as nanoyarn-enhanced collagen hydrogels, pose scalability challenges. Ensuring that these sophisticated materials can be produced consistently and at scale remains a significant hurdle.

The targeted delivery and controlled release of therapeutic agents within hydrogels continue to be areas needing improvement. HPS-fibrin hydrogels, for instance, require enhanced specificity for effective therapeutic delivery [51]. Hydrogels used in myocardial applications with SDF-1 and angiogenic peptides also need improved targeting to maximize their therapeutic potential [88].

## 18. Future Directions

Future research should focus on optimizing the mechanical properties of hydrogels to better mimic native tissue characteristics and enhance their performance in dynamic physiological environments. Combining different materials to create composite hydrogels, such as chitosan nanofibrous microspheres with PLGA-PEG-PLGA hydrogels, can provide enhanced mechanical stability and functionality [20,22,63,86,113,135].

Tailoring the degradation rates of hydrogels to precisely match the tissue regeneration process is crucial. This can be achieved through advanced crosslinking strategies and incorporating responsive elements that degrade under specific physiological conditions. Innovations in biodegradable polymers and crosslinking techniques, such as phenolic-chitosan self-healing hydrogels, may further be evaluated to achieve controlled and predictable degradation [52,65,81,118].

Developing scalable and cost-effective manufacturing techniques is essential for the clinical translation of injectable hydrogels. This includes simplifying synthesis processes and ensuring consistent quality control. Collaborative efforts between academia and industry can facilitate the development of standardized protocols and regulatory frameworks for the production of these advanced materials [37,80,95].

Incorporating targeting moieties and responsive elements into hydrogels can improve the specificity and efficiency of therapeutic delivery. This includes the use of smart materials that respond to environmental cues such as pH, temperature, or specific biomolecules. Research into novel delivery systems, like magnetically responsive hydrogels, can provide new avenues for targeted and controlled release applications in tissue engineering and regenerative medicine [18,47,88,129].

Combining injectable hydrogels with emerging technologies such as 3D bioprinting and nanotechnology can open new possibilities for creating complex tissue structures and enhancing regenerative outcomes. The development of bioinspired self-healing nanocomposite hydrogels, leveraging nanomaterials for improved mechanical properties and bioactivity, represents a promising direction for future research. Innovations like injectable biomimetic porous hydrogels with Mg^2+^ release and enhanced osteogenic differentiation, and nanoengineered hydrogels with chitosan, ACSCs, and mesoporous SiO_2_ nanoparticles, demonstrate the potential of integrating advanced materials science with biomedical engineering [14,61,80,115].

## 19. Conclusions

Injectable biomimetic gels have significantly advanced biomedical applications through their enhanced biocompatibility, multifunctionality, and controlled delivery efficacy. Despite these achievements, challenges related to mechanical resilience, degradation control, scalability, and targeted delivery remain. Future research should focus on optimizing mechanical properties, refining biodegradation rates, developing scalable manufacturing techniques, and enhancing targeted delivery systems. Integrating emerging technologies such as 3D bioprinting and nanotechnology will further enhance their therapeutic potential. Collaborative efforts between academia and industry are crucial to standardizing production and ensuring clinical translation. These strategies promise to address current limitations and unlock the full potential of injectable hydrogels in tissue engineering and regenerative medicine.

## References

[1] Hou M., Wang X., Yue O., Zheng M., Zhang H., Liu X. (2022). Development of a multifunctional injectable temperature-sensitive gelatin-based adhesive double-network hydrogel. Biomater. Adv..

[2] Liang Y., Xu H., Li Z., Zhangji A., Guo B. (2022). Bioinspired Injectable Self-Healing Hydrogel Sealant with Fault-Tolerant and Repeated Thermo-Responsive Adhesion for Sutureless Post-Wound-Closure and Wound Healing. Nano-Micro Lett..

[3] Liu Y., Meng H., Konst S., Sarmiento R., Rajachar R., Lee B.P. (2014). Injectable dopamine-modified poly(ethylene glycol) nanocomposite hydrogel with enhanced adhesive property and bioactivity. ACS Appl. Mater. Interfaces.

[4] Yan S., Wang W., Li X., Ren J., Yun W., Zhang K., Li G., Yin J. (2018). Preparation of mussel-inspired injectable hydrogels based on dual-functionalized alginate with improved adhesive, self-healing, and mechanical properties. J. Mat. Chem. B.

[5] Wang R., Liu L., He X., Xia Z., Zhao Z., Xi Z., Yu J., Wang J. (2023). Dynamic Crosslinked Injectable Mussel-Inspired Hydrogels with Adhesive, Self-Healing, and Biodegradation Properties. Polymers.

[6] Sanmartín-Masiá E., Poveda-Reyes S., Gallego Ferrer G. (2016). Extracellular matrix–inspired gelatin/hyaluronic acid injectable hydrogels. Int. J. Polym. Mater. Polym. Biomater..

[7] Pescosolido L., Miatto S., Di Meo C., Cencetti C., Coviello T., Alhaique F., Matricardi P. (2010). Injectable and in situ gelling hydrogels for modified protein release. Eur. Biophys. J..

[8] Karami P., Nasrollahzadeh N., Wyss C., O’Sullivan A., Broome M., Procter P., Bourban P.E., Moser C., Pioletti D.P. (2021). An Intrinsically-Adhesive Family of Injectable and Photo-Curable Hydrogels with Functional Physicochemical Performance for Regenerative Medicine. Macromol. Rapid Commun..

[9] Shi Z., Zhong Q., Chen Y., Gao J., Pan X., Lian Q., Chen R., Wang P., Wang J., Shi Z. (2021). Nanohydroxyapatite, Nanosilicate-Reinforced Injectable, and Biomimetic Gelatin-Methacryloyl Hydrogel for Bone Tissue Engineering. Int. J. Nanomed..

[10] Bonetti L., Borsacchi S., Soriente A., Boccali A., Calucci L., Raucci M.G., Altomare L. (2024). Injectable in situ gelling methylcellulose-based hydrogels for bone tissue regeneration. J. Mat. Chem. B.

[11] Thambi T., Phan V.H., Lee D.S. (2016). Stimuli-Sensitive Injectable Hydrogels Based on Polysaccharides and Their Biomedical Applications. Macromol. Rapid Commun..

[12] Fu H., Yu C., Li X., Bao H., Zhang B., Chen Z., Zhang Z. (2021). Facile engineering of ECM-mimetic injectable dual crosslinking hydrogels with excellent mechanical resilience, tissue adhesion, and biocompatibility. J. Mat. Chem. B.

[13] Kim S.H., Thambi T., Giang Phan V.H., Lee D.S. (2020). Modularly engineered alginate bioconjugate hydrogel as biocompatible injectable scaffold for in situ biomineralization. Carbohydr. Polym..

[14] Ma W., Yang M., Wu C., Wang S., Du M. (2023). Bioinspired self-healing injectable nanocomposite hydrogels based on oxidized dextran and gelatin for growth-factor-free bone regeneration. Int. J. Biol. Macromol..

[15] Yao S., Xu Y., Zhou Y., Shao C., Liu Z., Jin B., Zhao R., Cao H., Pan H., Tang R. (2019). Calcium Phosphate Nanocluster-Loaded Injectable Hydrogel for Bone Regeneration. ACS Appl. Bio Mater..

[16] Naranda J., Bracic M., Vogrin M., Maver U. (2021). Recent Advancements in 3D Printing of Polysaccharide Hydrogels in Cartilage Tissue Engineering. Materials.

[17] Wang L.L., Chen Z.J., Yan Y.F., He C., Li X.M. (2021). Fabrication of injectable hydrogels from silk fibroin and angiogenic peptides for vascular growth and tissue regeneration. Chem. Eng. J..

[18] Araujo-Custodio S., Gomez-Florit M., Tomas A.R., Mendes B.B., Babo P.S., Mithieux S.M., Weiss A., Domingues R.M.A., Reis R.L., Gomes M.E. (2019). Injectable and Magnetic Responsive Hydrogels with Bioinspired Ordered Structures. ACS Biomater. Sci. Eng..

[19] Nageeb M., Nouh S.R., Bergman K., Nagy N.B., Khamis D., Kisiel M., Engstrand T., Hilborn J., Marei M.K. (2012). Bone Engineering by Biomimetic Injectable Hydrogel. Mol. Cryst. Liq. Cryst..

[20] Han S., Yang H., Ni X., Deng Y., Li Z., Xing X., Du M. (2023). Programmed release of vascular endothelial growth factor and exosome from injectable chitosan nanofibrous microsphere-based PLGA-PEG-PLGA hydrogel for enhanced bone regeneration. Int. J. Biol. Macromol..

[21] Wasupalli G.K., Verma D. (2022). Thermosensitive injectable hydrogel based on chitosan-polygalacturonic acid polyelectrolyte complexes for bone tissue engineering. Carbohydr. Polym..

[22] Wang M., Feng X.W., Wang T.F., Gao Y.X., Wang Y.N., Sa Y., Jiang T. (2016). Synthesis and characterization of an injectable and self-curing poly(methyl methacrylate) cement functionalized with a biomimetic chitosan-poly(vinyl alcohol)/nano-sized hydroxyapatite/silver hydrogel. Rsc Adv..

[23] Wang Y., Peng Z., Zhang D., Song D. (2023). Tough, Injectable Calcium Phosphate Cement Based Composite Hydrogels to Promote Osteogenesis. Gels.

[24] Balitaan J.N.I., Luo W.J., Su Y.W., Yu C.Y., Wu T.Y., Chang C.A., Jia H.W., Lin S.R., Hsiao C.D., Yeh J.M. (2023). Healing Wounds Efficiently with Biomimetic Soft Matter: Injectable Self-Healing Neutral Glycol Chitosan/Dibenzaldehyde-Terminated Poly(ethylene glycol) Hydrogel with Inherent Antibacterial Properties. ACS Appl. Bio Mater..

[25] Liu X., Zhang Y., Liu Y., Hua S., Meng F., Ma Q., Kong L., Pan S., Che Y. (2023). Injectable, self-healable and antibacterial multi-responsive tunicate cellulose nanocrystals strengthened supramolecular hydrogels for wound dressings. Int. J. Biol. Macromol..

[26] Xiong L., Zhou C., Tong L., Han X., Zou Y., Dong Z., Liang J., Chen Y., Fan Y. (2023). Injectable hydrogels of recombinant human collagen type III and chitosan with antibacterial and antioxidative activities for wound healing. J. Mat. Chem. B.

[27] Le T.M.D., Duong H.T.T., Thambi T., Giang Phan V.H., Jeong J.H., Lee D.S. (2018). Bioinspired pH- and Temperature-Responsive Injectable Adhesive Hydrogels with Polyplexes Promotes Skin Wound Healing. Biomacromolecules.

[28] Chen T., Wang Y., Xie J., Qu X., Liu C. (2022). Lysozyme Amyloid Fibril-Integrated PEG Injectable Hydrogel Adhesive with Improved Antiswelling and Antibacterial Capabilities. Biomacromolecules.

[29] Chen X., Du D., Zhang Z., Shi C., Hua Z., Chen J., Shi D. (2023). Injectable Dopamine-Polysaccharide In Situ Composite Hydrogels with Enhanced Adhesiveness. ACS Biomater. Sci. Eng..

[30] Omidian H., Chowdhury S.D. (2023). Advancements and Applications of Injectable Hydrogel Composites in Biomedical Research and Therapy. Gels.

[31] Omidian H., Dey Chowdhury S. (2024). Swellable Microneedles in Drug Delivery and Diagnostics. Pharmaceuticals.

[32] Omidian H., Park K., Ottenbrite R.M., Park K., Okano T. (2010). Introduction to Hydrogels. Biomedical Applications of Hydrogels Handbook.

[33] Omidian H., Park K., Siepmann J., Siegel R.A., Rathbone M.J. (2012). Hydrogels. Fundamentals and Applications of Controlled Release Drug Delivery.

[34] Omidian H., Wilson R.L., Gill E.J. (2024). Advancements and Challenges in Self-Healing Hydrogels for Wound Care. Gels.

[35] Zhang R., Lin M., Wang C., Li Y., Li Y., Zou Q. (2022). Bioinspired fabrication of EDC-crosslinked gelatin/nanohydroxyapatite injectable microspheres for bone repair. Int. J. Polym. Mater. Polym. Biomater..

[36] Jiang X., Zeng F., Zhang L., Yu A., Lu A. (2023). Engineered Injectable Cell-Laden Chitin/Chitosan Hydrogel with Adhesion and Biodegradability for Calvarial Defect Regeneration. ACS Appl. Mater. Interfaces.

[37] Liu W., Zhan J., Su Y., Wu T., Ramakrishna S., Liao S., Mo X. (2014). Injectable hydrogel incorporating with nanoyarn for bone regeneration. J. Biomater. Sci. Polym. Ed..

[38] Lewandowska-Lancucka J., Gilarska A., Bula A., Horak W., Latkiewicz A., Nowakowska M. (2019). Genipin crosslinked bioactive collagen/chitosan/hyaluronic acid injectable hydrogels structurally amended via covalent attachment of surface-modified silica particles. Int. J. Biol. Macromol..

[39] Ma L., Su W., Ran Y., Ma X., Yi Z., Chen G., Chen X., Deng Z., Tong Q., Wang X. (2020). Synthesis and characterization of injectable self-healing hydrogels based on oxidized alginate-hybrid-hydroxyapatite nanoparticles and carboxymethyl chitosan. Int. J. Biol. Macromol..

[40] Balakrishnan B., Joshi N., Jayakrishnan A., Banerjee R. (2014). Self-crosslinked oxidized alginate/gelatin hydrogel as injectable, adhesive biomimetic scaffolds for cartilage regeneration. Acta Biomater..

[41] Yan S., Wang T., Feng L., Zhu J., Zhang K., Chen X., Cui L., Yin J. (2014). Injectable in situ self-cross-linking hydrogels based on poly(L-glutamic acid) and alginate for cartilage tissue engineering. Biomacromolecules.

[42] Wang L., Dong S., Liu Y., Ma Y., Zhang J., Yang Z., Jiang W., Yuan Y. (2020). Fabrication of Injectable, Porous Hyaluronic Acid Hydrogel Based on an In-Situ Bubble-Forming Hydrogel Entrapment Process. Polymers.

[43] Chen J.P., Tsai M.J., Liao H.T. (2013). Incorporation of biphasic calcium phosphate microparticles in injectable thermoresponsive hydrogel modulates bone cell proliferation and differentiation. Colloids Surf. B Biointerfaces.

[44] Chen F., Ni Y., Liu B., Zhou T., Yu C., Su Y., Zhu X., Yu X., Zhou Y. (2017). Self-crosslinking and injectable hyaluronic acid/RGD-functionalized pectin hydrogel for cartilage tissue engineering. Carbohydr. Polym..

[45] Meng L., Shao C., Cui C., Xu F., Lei J., Yang J. (2020). Autonomous Self-Healing Silk Fibroin Injectable Hydrogels Formed via Surfactant-Free Hydrophobic Association. ACS Appl. Mater. Interfaces.

[46] Ding Z., Han H., Fan Z., Lu H., Sang Y., Yao Y., Cheng Q., Lu Q., Kaplan D.L. (2017). Nanoscale Silk-Hydroxyapatite Hydrogels for Injectable Bone Biomaterials. ACS Appl. Mater. Interfaces.

[47] Luo S., Wu J., Jia Z., Tang P., Sheng J., Xie C., Liu C., Gan D., Hu D., Zheng W. (2019). An Injectable, Bifunctional Hydrogel with Photothermal Effects for Tumor Therapy and Bone Regeneration. Macromol. Biosci..

[48] Yang T., Xie P., Wu Z., Liao Y., Chen W., Hao Z., Wang Y., Zhu Z., Teng W. (2020). The Injectable Woven Bone-Like Hydrogel to Perform Alveolar Ridge Preservation with Adapted Remodeling Performance After Tooth Extraction. Front. Bioeng. Biotechnol..

[49] Liu S.Q., Tian Q., Wang L., Hedrick J.L., Hui J.H., Yang Y.Y., Ee P.L. (2010). Injectable Biodegradable Poly(ethylene glycol)/RGD Peptide Hybrid Hydrogels for in vitro Chondrogenesis of Human Mesenchymal Stem Cells. Macromol. Rapid Commun..

[50] Pena B., Bosi S., Aguado B.A., Borin D., Farnsworth N.L., Dobrinskikh E., Rowland T.J., Martinelli V., Jeong M., Taylor M.R.G. (2017). Injectable Carbon Nanotube-Functionalized Reverse Thermal Gel Promotes Cardiomyocytes Survival and Maturation. ACS Appl. Mater. Interfaces.

[51] Hadjipanayi E., Moog P., Bekeran S., Kirchhoff K., Berezhnoi A., Aguirre J., Bauer A.T., Kukrek H., Schmauss D., Hopfner U. (2019). In Vitro Characterization of Hypoxia Preconditioned Serum (HPS)-Fibrin Hydrogels: Basis for an Injectable Biomimetic Tissue Regeneration Therapy. J. Funct. Biomater..

[52] Lin S.H., Papadakis C.M., Kang J.J., Lin J.M., Hsu S.H. (2021). Injectable Phenolic-Chitosan Self-Healing Hydrogel with Hierarchical Micelle Architectures and Fast Adhesiveness. Chem. Mater..

[53] Khan M., Koivisto J.T., Hukka T.I., Hokka M., Kellomaki M. (2018). Composite Hydrogels Using Bioinspired Approach with in Situ Fast Gelation and Self-Healing Ability as Future Injectable Biomaterial. ACS Appl. Mater. Interfaces.

[54] Chen Y., Wang W., Wu D., Nagao M., Hall D.G., Thundat T., Narain R. (2018). Injectable Self-Healing Zwitterionic Hydrogels Based on Dynamic Benzoxaborole-Sugar Interactions with Tunable Mechanical Properties. Biomacromolecules.

[55] Tan Y., Ma L., Chen X., Ran Y., Tong Q., Tang L., Li X. (2022). Injectable hyaluronic acid/hydroxyapatite composite hydrogels as cell carriers for bone repair. Int. J. Biol. Macromol..

[56] Cheng W., Ding Z., Zheng X., Lu Q., Kong X., Zhou X., Lu G., Kaplan D.L. (2020). Injectable hydrogel systems with multiple biophysical and biochemical cues for bone regeneration. Biomater. Sci..

[57] Griffanti G., Jiang W., Nazhat S.N. (2019). Bioinspired mineralization of a functionalized injectable dense collagen hydrogel through silk sericin incorporation. Biomater. Sci..

[58] Gilarska A., Lewandowska-Lancucka J., Horak W., Nowakowska M. (2018). Collagen/chitosan/hyaluronic acid—Based injectable hydrogels for tissue engineering applications—Design, physicochemical and biological characterization. Colloid. Surf. B-Biointerfaces.

[59] Liu X., Zhang Y., Hussain Z., Zheng P., Xu M., Zhao H., Liu Y., Cao Y., Ullah I., Osaka A. (2023). Self-biomineralized in situ injectable CaSO4 nanorods-enriched collagen-hyaluronic acid composite hydrogels for biomimetic bone reconstruction in a minimally invasive manner. Appl. Mater. Today.

[60] Fu S., Ni P., Wang B., Chu B., Zheng L., Luo F., Luo J., Qian Z. (2012). Injectable and thermo-sensitive PEG-PCL-PEG copolymer/collagen/n-HA hydrogel composite for guided bone regeneration. Biomaterials.

[61] Zhou H., Yu K., Jiang H., Deng R., Chu L., Cao Y., Zheng Y., Lu W., Deng Z., Liang B. (2021). A Three-in-One Strategy: Injectable Biomimetic Porous Hydrogels for Accelerating Bone Regeneration via Shape-Adaptable Scaffolds, Controllable Magnesium Ion Release, and Enhanced Osteogenic Differentiation. Biomacromolecules.

[62] Chen X., Yan J., Jiang Y., Fan Y., Ying Z., Tan S., Zhou Z., Liu J., Chen F., He S. (2022). Platelet-Activating Biominerals Enhanced Injectable Hydrogels with Superior Bioactivity for Bone Regeneration. Front. Bioeng. Biotechnol..

[63] Sa Y., Wang M., Deng H.B., Wang Y.N., Jiang T. (2015). Beneficial effects of biomimetic nano-sized hydroxyapatite/antibiotic gentamicin enriched chitosan-glycerophosphate hydrogel on the performance of injectable polymethylmethacrylate. Rsc Adv..

[64] Liu B., Li J., Lei X., Miao S., Zhang S., Cheng P., Song Y., Wu H., Gao Y., Bi L. (2020). Cell-loaded injectable gelatin/alginate/LAPONITE(R) nanocomposite hydrogel promotes bone healing in a critical-size rat calvarial defect model. RSC Adv..

[65] Li Y.M., Xiao L., Wei D.X., Liu S.Y., Zhang Z.R., Lian R.X., Wang L.R., Chen Y.S., Jiang J., Xiao Y. (2023). Injectable Biomimetic Hydrogel Guided Functional Bone Regeneration by Adapting Material Degradation to Tissue Healing. Adv. Funct. Mater..

[66] Behan K., Dufour A., Garcia O., Kelly D. (2022). Methacrylated Cartilage ECM-Based Hydrogels as Injectables and Bioinks for Cartilage Tissue Engineering. Biomolecules.

[67] Yu H., Feng M., Mao G., Li Q., Zhang Z., Bian W., Qiu Y. (2022). Implementation of Photosensitive, Injectable, Interpenetrating, and Kartogenin-Modified GELMA/PEDGA Biomimetic Scaffolds to Restore Cartilage Integrity in a Full-Thickness Osteochondral Defect Model. ACS Biomater. Sci. Eng..

[68] Qu Y., He S., Luo S., Zhao J., Liang R., Liao C., Zheng L. (2023). Photocrosslinkable, Injectable Locust Bean Gum Hydrogel Induces Chondrogenic Differentiation of Stem Cells for Cartilage Regeneration. Adv. Healthc. Mater..

[69] Qi C., Liu J., Jin Y., Xu L., Wang G., Wang Z., Wang L. (2018). Photo-crosslinkable, injectable sericin hydrogel as 3D biomimetic extracellular matrix for minimally invasive repairing cartilage. Biomaterials.

[70] Jin R., Teixeira L.S., Dijkstra P.J., van Blitterswijk C.A., Karperien M., Feijen J. (2010). Enzymatically-crosslinked injectable hydrogels based on biomimetic dextran-hyaluronic acid conjugates for cartilage tissue engineering. Biomaterials.

[71] Kim H.S., Li C.J., Park S.M., Kim K.W., Mo J.H., Jin G.Z., Lee H.H., Kim H.W., Shin U.S., Lee J.H. (2024). Development of an Injectable Biphasic Hyaluronic Acid-Based Hydrogel with Stress Relaxation Properties for Cartilage Regeneration. Adv. Healthc. Mater..

[72] Yao Y., Wang P., Li X., Xu Y., Lu G., Jiang Q., Sun Y., Fan Y., Zhang X. (2020). A di-self-crosslinking hyaluronan-based hydrogel combined with type I collagen to construct a biomimetic injectable cartilage-filling scaffold. Acta Biomater..

[73] Chen F., Yu S., Liu B., Ni Y., Yu C., Su Y., Zhu X., Yu X., Zhou Y., Yan D. (2016). An Injectable Enzymatically Crosslinked Carboxymethylated Pullulan/Chondroitin Sulfate Hydrogel for Cartilage Tissue Engineering. Sci. Rep..

[74] Li X., Xu Q., Johnson M., Wang X., Lyu J., Li Y., McMahon S., Greiser U., Sigen A., Wang W. (2021). A chondroitin sulfate based injectable hydrogel for delivery of stem cells in cartilage regeneration. Biomater. Sci..

[75] Li T., Song X.B., Weng C.M., Wang X., Sun L., Gong X.Y., Yang L., Chen C. (2018). Self-crosslinking and injectable chondroitin sulfate/pullulan hydrogel for cartilage tissue engineering. Appl. Mater. Today.

[76] Gao Y., Li B., Kong W., Yuan L., Guo L., Li C., Fan H., Fan Y., Zhang X. (2018). Injectable and self-crosslinkable hydrogels based on collagen type II and activated chondroitin sulfate for cell delivery. Int. J. Biol. Macromol..

[77] Cao Y., Zhang H., Qiu M., Zheng Y., Shi X., Yang J. (2024). Biomimetic injectable and bilayered hydrogel scaffold based on collagen and chondroitin sulfate for the repair of osteochondral defects. Int. J. Biol. Macromol..

[78] Dixit A., Mahajan A., Saxena R., Chakraborty S., Katti D.S. (2024). Engineering sulfated polysaccharides and silk fibroin based injectable IPN hydrogels with stiffening and growth factor presentation abilities for cartilage tissue engineering. Biomater. Sci..

[79] Li H., Tong Z., Fang Y., Liu F., He F., Teng C. (2024). Biomimetic Injectable Hydrogel Based on Methacrylate-Modified Silk Fibroin Embedded with Kartogenin for Superficial Cartilage Regeneration. ACS Biomater. Sci. Eng..

[80] Cui P., Pan P., Qin L., Wang X., Chen X., Deng Y., Zhang X. (2023). Nanoengineered hydrogels as 3D biomimetic extracellular matrix with injectable and sustained delivery capability for cartilage regeneration. Bioact. Mater..

[81] Wang J., Li B., Pu X., Wang X., Cooper R.C., Gui Q., Yang H. (2020). Injectable Multicomponent Biomimetic Gel Composed of Inter-Crosslinked Dendrimeric and Mesoporous Silica Nanoparticles Exhibits Highly Tunable Elasticity and Dual Drug Release Capacity. ACS Appl. Mater. Interfaces.

[82] Ren K., He C., Xiao C., Li G., Chen X. (2015). Injectable glycopolypeptide hydrogels as biomimetic scaffolds for cartilage tissue engineering. Biomaterials.

[83] Gudde A.N., van Velthoven M.J.J., Kouwer P.H.J., Roovers J.W.R., Guler Z. (2023). Injectable polyisocyanide hydrogel as healing supplement for connective tissue regeneration in an abdominal wound model. Biomaterials.

[84] Phan V.H.G., Murugesan M., Nguyen P.P.T., Luu C.H., Le N.H., Nguyen H.T., Manivasagan P., Jang E.S., Li Y., Thambi T. (2022). Biomimetic injectable hydrogel based on silk fibroin/hyaluronic acid embedded with methylprednisolone for cartilage regeneration. Colloids Surf. B Biointerfaces.

[85] Qi J., Yan Y., Cheng B., Deng L., Shao Z., Sun Z., Li X. (2018). Enzymatic Formation of an Injectable Hydrogel from a Glycopeptide as a Biomimetic Scaffold for Vascularization. ACS Appl. Mater. Interfaces.

[86] Jeencham R., Sinna J., Ruksakulpiwat C., Tawonsawatruk T., Numpaisal P.O., Ruksakulpiwat Y. (2024). Development of Biphasic Injectable Hydrogels for Meniscus Scaffold from Photocrosslinked Glycidyl Methacrylate-Modified Poly(Vinyl Alcohol)/Glycidyl Methacrylate-Modified Silk Fibroin. Polymers.

[87] Meng X., Stout D.A., Sun L., Beingessner R.L., Fenniri H., Webster T.J. (2013). Novel injectable biomimetic hydrogels with carbon nanofibers and self assembled rosette nanotubes for myocardial applications. J. Biomed. Mater. Res. A.

[88] Song M., Jang H., Lee J., Kim J.H., Kim S.H., Sun K., Park Y. (2014). Regeneration of chronic myocardial infarction by injectable hydrogels containing stem cell homing factor SDF-1 and angiogenic peptide Ac-SDKP. Biomaterials.

[89] Fang W., Yang L., Chen Y., Hu Q. (2023). Bioinspired multifunctional injectable hydrogel for hemostasis and infected wound management. Acta Biomater..

[90] Gagana Rao R., Kumar A.S., Prema D., Prakash J., Balashanmugam P., Devanand Venkatasubbu G. (2024). GO/CaCO_3_/SiO_2_ nanocomposite incorporated Carrageenan/Chitosan injectable hydrogel for enhanced hemostasis. Inorg. Chem. Commun..

[91] Zhou F., Yang Y., Zhang W., Liu S., Shaikh A.B., Yang L., Lai Y., Ouyang H., Zhu W. (2022). Bioinspired, injectable, tissue-adhesive and antibacterial hydrogel for multiple tissue regeneration by minimally invasive therapy. Appl. Mater. Today.

[92] Wei K., Senturk B., Matter M.T., Wu X., Herrmann I.K., Rottmar M., Toncelli C. (2019). Mussel-Inspired Injectable Hydrogel Adhesive Formed under Mild Conditions Features Near-Native Tissue Properties. ACS Appl. Mater. Interfaces.

[93] Deng Z.W., Li M.H., Hu Y., He Y., Tao B.L., Yuan Z., Wang R., Chen M.W., Luo Z., Cai K.Y. (2021). Injectable biomimetic hydrogels encapsulating Gold/metal-organic frameworks nanocomposites for enhanced antibacterial and wound healing activity under visible light actuation. Chem. Eng. J..

[94] Chen K., Wu Z., Liu Y., Yuan Y., Liu C. (2021). Injectable Double-Crosslinked Adhesive Hydrogels with High Mechanical Resilience and Effective Energy Dissipation for Joint Wound Treatment. Adv. Funct. Mater..

[95] Ghosh S., Ghosh T., Bhowmik S., Patidar M.K., Das A.K. (2023). Nucleopeptide-Coupled Injectable Bioconjugated Guanosine-Quadruplex Hydrogel with Inherent Antibacterial Activity. ACS Appl. Bio Mater..

[96] Li S., Li X., Xu Y., Fan C., Li Z.A., Zheng L., Luo B., Li Z.P., Lin B., Zha Z.G. (2024). Collagen fibril-like injectable hydrogels from self-assembled nanoparticles for promoting wound healing. Bioact. Mater..

[97] Wang C., Niu H., Ma X., Hong H., Yuan Y., Liu C. (2019). Bioinspired, Injectable, Quaternized Hydroxyethyl Cellulose Composite Hydrogel Coordinated by Mesocellular Silica Foam for Rapid, Noncompressible Hemostasis and Wound Healing. ACS Appl. Mater. Interfaces.

[98] Zhang D., Wang B., Sun Y., Wang C., Mukherjee S., Yang C., Chen Y. (2020). Injectable Enzyme-Based Hydrogel Matrix with Precisely Oxidative Stress Defense for Promoting Dermal Repair of Burn Wound. Macromol. Biosci..

[99] Zhou S., Li L., Chen C., Chen Y., Zhou L., Zhou F.H., Dong J., Wang L. (2021). Injectable gelatin microspheres loaded with platelet rich plasma improve wound healing by regulating early inflammation. Int. J. Med. Sci..

[100] Han Y., Yang J., Zhao W., Wang H., Sun Y., Chen Y., Luo J., Deng L., Xu X., Cui W. (2021). Biomimetic injectable hydrogel microspheres with enhanced lubrication and controllable drug release for the treatment of osteoarthritis. Bioact. Mater..

[101] Zhou J.K., Zhang K., Ma S.S., Liu T.F., Yao M.H., Li J.G., Wang X.F., Guan F.X. (2019). Preparing an injectable hydrogel with sodium alginate and Type I collagen to create better MSCs growth microenvironment. e-Polymers.

[102] Bhattacharjee M., Escobar Ivirico J.L., Kan H.M., Shah S., Otsuka T., Bordett R., Barajaa M., Nagiah N., Pandey R., Nair L.S. (2022). Injectable amnion hydrogel-mediated delivery of adipose-derived stem cells for osteoarthritis treatment. Proc. Natl. Acad. Sci. USA.

[103] Yang R., Tan L.H., Cen L., Zhang Z.B. (2016). An injectable scaffold based on crosslinked hyaluronic acid gel for tissue regeneration. Rsc Adv..

[104] Mulyasasmita W., Cai L., Dewi R.E., Jha A., Ullmann S.D., Luong R.H., Huang N.F., Heilshorn S.C. (2014). Avidity-controlled hydrogels for injectable co-delivery of induced pluripotent stem cell-derived endothelial cells and growth factors. J. Control. Release.

[105] Li J., Marmorat C., Vasilyev G., Jiang J., Koifman N., Guo Y., Talmon I., Zussman E., Gersappe D., Davis R. (2019). Flow induced stability of pluronic hydrogels: Injectable and unencapsulated nucleus pulposus replacement. Acta Biomater..

[106] Young D.A., Ibrahim D.O., Hu D., Christman K.L. (2011). Injectable hydrogel scaffold from decellularized human lipoaspirate. Acta Biomater..

[107] Furlani F., Montanari M., Sangiorgi N., Saracino E., Campodoni E., Sanson A., Benfenati V., Tampieri A., Panseri S., Sandri M. (2022). Electroconductive and injectable hydrogels based on gelatin and PEDOT:PSS for a minimally invasive approach in nervous tissue regeneration. Biomater. Sci..

[108] Farrell K., Joshi J., Kothapalli C.R. (2017). Injectable uncrosslinked biomimetic hydrogels as candidate scaffolds for neural stem cell delivery. J. Biomed. Mater. Res. A.

[109] Hasanzadeh E., Seifalian A., Mellati A., Saremi J., Asadpour S., Enderami S.E., Nekounam H., Mahmoodi N. (2023). Injectable hydrogels in central nervous system: Unique and novel platforms for promoting extracellular matrix remodeling and tissue engineering. Mater. Today Bio.

[110] Sivan S.S., Roberts S., Urban J.P., Menage J., Bramhill J., Campbell D., Franklin V.J., Lydon F., Merkher Y., Maroudas A. (2014). Injectable hydrogels with high fixed charge density and swelling pressure for nucleus pulposus repair: Biomimetic glycosaminoglycan analogues. Acta Biomater..

[111] Duan N., Mei L., Hu L., Yin X., Wei X., Li Y., Li Q., Zhao G., Zhou Q., Du Z. (2023). Biomimetic, Injectable, and Self-Healing Hydrogels with Sustained Release of Ranibizumab to Treat Retinal Neovascularization. ACS Appl. Mater. Interfaces.

[112] Eltawila A.M., Hassan M.N., Safaan S.M., Abd El-Fattah A., Zakaria O., El-Khordagui L.K., Kandil S. (2021). Local treatment of experimental mandibular osteomyelitis with an injectable biomimetic gentamicin hydrogel using a new rabbit model. J. Biomed. Mater. Res. Part. B.

[113] Mahajan A., Singh A., Datta D., Katti D.S. (2022). Bioinspired Injectable Hydrogels Dynamically Stiffen and Contract to Promote Mechanosensing-Mediated Chondrogenic Commitment of Stem Cells. ACS Appl. Mater. Interfaces.

[114] Kumar M., Gupta P., Bhattacharjee S., Nandi S.K., Mandal B.B. (2018). Immunomodulatory injectable silk hydrogels maintaining functional islets and promoting anti-inflammatory M2 macrophage polarization. Biomaterials.

[115] De France K.J., Yager K.G., Chan K.J.W., Corbett B., Cranston E.D., Hoare T. (2017). Injectable Anisotropic Nanocomposite Hydrogels Direct in Situ Growth and Alignment of Myotubes. Nano Lett..

[116] Li P., Li Y., Fu R., Duan Z., Zhu C., Fan D. (2023). NIR- and pH-responsive injectable nanocomposite alginate-graft-dopamine hydrogel for melanoma suppression and wound repair. Carbohydr. Polym..

[117] Joshi Navare K., Colombani T., Rezaeeyazdi M., Bassous N., Rana D., Webster T., Memic A., Bencherif S.A. (2020). Needle-injectable microcomposite cryogel scaffolds with antimicrobial properties. Sci. Rep..

[118] Ouro P.M.S., Costa D.C.S., Amaral A.J.R., Mano J.F. (2024). A Supramolecular Injectable Methacryloyl Chitosan-Tricine-Based Hydrogel with 3D Printing Potential for Tissue Engineering Applications. Macromol. Biosci..

[119] Waduthanthri K.D., He Y., Montemagno C., Cetinel S. (2019). An injectable peptide hydrogel for reconstruction of the human trabecular meshwork. Acta Biomater..

[120] Thomas J.A., Hinton Z.R., Korley L.T.J. (2023). Peptide-polyurea hybrids: A platform for tunable, thermally-stable, and injectable hydrogels. Soft Matter.

[121] Borrelli C., Buckley C.T. (2020). Injectable Disc-Derived ECM Hydrogel Functionalised with Chondroitin Sulfate for Intervertebral Disc Regeneration. Acta Biomater..

[122] Xu Q., Zhang Z., Xiao C., He C., Chen X. (2017). Injectable Polypeptide Hydrogel as Biomimetic Scaffolds with Tunable Bioactivity and Controllable Cell Adhesion. Biomacromolecules.

[123] Bardill J., Williams S.M., Shabeka U., Niswander L., Park D., Marwan A.I. (2019). An Injectable Reverse Thermal Gel for Minimally Invasive Coverage of Mouse Myelomeningocele. J. Surg. Res..

[124] Li R., Lyu Y., Luo S., Wang H., Zheng X., Li L., Ao N., Zha Z. (2021). Fabrication of a multi-level drug release platform with liposomes, chitooligosaccharides, phospholipids and injectable chitosan hydrogel to enhance anti-tumor effectiveness. Carbohydr. Polym..

[125] Zhang Z., Li A., Min X., Zhang Q., Yang J., Chen G., Zou M., Sun W., Cheng G. (2021). An ROS-sensitive tegafur-PpIX-heterodimer-loaded in situ injectable thermosensitive hydrogel for photodynamic therapy combined with chemotherapy to enhance the tegafur-based treatment of breast cancer. Biomater. Sci..

[126] Parsana N., Seoud O.E., Al-Ghamdi A., Kasoju N., Malek N. (2024). Deep Eutectic Solvent and Poly (Vinyl Alcohol) Based Self-healable, Injectable and Adhesive „Eutectogel”: An Emerging Drug Delivery Vehicle. ChemistrySelect.

[127] Selvam A., Majood M., Chaurasia R., Rupesh, Singh A., Dey T., Agrawal O., Verma Y.K., Mukherjee M. (2023). Injectable organo-hydrogels influenced by click chemistry as a paramount stratagem in the conveyor belt of pharmaceutical revolution. J. Mat. Chem. B.

[128] Wang T., Ding J., Chen Z., Zhang Z., Rong Y., Li G., He C., Chen X. (2024). Injectable, Adhesive Albumin Nanoparticle-Incorporated Hydrogel for Sustained Localized Drug Delivery and Efficient Tumor Treatment. ACS Appl. Mater. Interfaces.

[129] Jin M.Y., Gläser A., Paez J.I. (2022). Redox-triggerable firefly luciferin-bioinspired hydrogels as injectable and cell-encapsulating matrices. Polym. Chem..

[130] Chen L., Li L., Mo Q., Zhang X., Chen C., Wu Y., Zeng X., Deng K., Liu N., Zhu P. (2023). An injectable gelatin/sericin hydrogel loaded with human umbilical cord mesenchymal stem cells for the treatment of uterine injury. Bioeng. Transl. Med..

[131] Rodell C.B., Wade R.J., Purcell B.P., Dusaj N.N., Burdick J.A. (2015). Selective Proteolytic Degradation of Guest-Host Assembled, Injectable Hyaluronic Acid Hydrogels. ACS Biomater. Sci. Eng..

[132] Weiden J., Voerman D., Dolen Y., Das R.K., van Duffelen A., Hammink R., Eggermont L.J., Rowan A.E., Tel J., Figdor C.G. (2018). Injectable Biomimetic Hydrogels as Tools for Efficient T Cell Expansion and Delivery. Front. Immunol..

[133] Zhang Y., Yang J., Zhang J., Li S., Zheng L., Zhang Y., Meng H., Zhang X., Wu Z. (2020). A bio-inspired injectable hydrogel as a cell platform for real-time glycaemic regulation. J. Mat. Chem. B.

[134] Xiao L., Xie P., Ma J., Shi K., Dai Y., Pang M., Luo J., Tan Z., Ma Y., Wang X. (2023). A Bioinspired Injectable, Adhesive, and Self-Healing Hydrogel with Dual Hybrid Network for Neural Regeneration after Spinal Cord Injury. Adv. Mater..

[135] Taheri S., Bao G., He Z., Mohammadi S., Ravanbakhsh H., Lessard L., Li J., Mongeau L. (2022). Injectable, Pore-Forming, Perfusable Double-Network Hydrogels Resilient to Extreme Biomechanical Stimulations. Adv. Sci..

[136] Han H.W., Hou Y.T., Hsu S.H. (2019). Angiogenic potential of co-spheroids of neural stem cells and endothelial cells in injectable gelatin-based hydrogel. Mater. Sci. Eng. C-Mater. Biol. Appl..

[137] Ghandforoushan P., Alehosseini M., Golafshan N., Castilho M., Dolatshahi-Pirouz A., Hanaee J., Davaran S., Orive G. (2023). Injectable hydrogels for cartilage and bone tissue regeneration: A review. Int. J. Biol. Macromol..

